# Oligodendrocyte Neurofascin Independently Regulates Both Myelin Targeting and Sheath Growth in the CNS

**DOI:** 10.1016/j.devcel.2019.10.016

**Published:** 2019-12-16

**Authors:** Anna Klingseisen, Ana-Maria Ristoiu, Linde Kegel, Diane L. Sherman, Maria Rubio-Brotons, Rafael G. Almeida, Sigrid Koudelka, Silvia K. Benito-Kwiecinski, Richard J. Poole, Peter J. Brophy, David A. Lyons

**Affiliations:** 1 Centre for Discovery Brain Sciences, University of Edinburgh, Edinburgh EH16 4SB, UK; 2 Department of Cell and Developmental Biology, University College London, London WC1E 6BT, UK

**Keywords:** CNS myelination, oligodendrocyte, zebrafish, mouse, *in vivo* imaging, Neurofascin, Caspr

## Abstract

Selection of the correct targets for myelination and regulation of myelin sheath growth are essential for central nervous system (CNS) formation and function. Through a genetic screen in zebrafish and complementary analyses in mice, we find that loss of oligodendrocyte Neurofascin leads to mistargeting of myelin to cell bodies, without affecting targeting to axons. In addition, loss of Neurofascin reduces CNS myelination by impairing myelin sheath growth. Time-lapse imaging reveals that the distinct myelinating processes of individual oligodendrocytes can engage in target selection and sheath growth at the same time and that Neurofascin concomitantly regulates targeting and growth. Disruption to Caspr, the neuronal binding partner of oligodendrocyte Neurofascin, also impairs myelin sheath growth, likely reflecting its association in an adhesion complex at the axon-glial interface with Neurofascin. Caspr does not, however, affect myelin targeting, further indicating that Neurofascin independently regulates distinct aspects of CNS myelination by individual oligodendrocytes *in vivo*.

## Highlights

• Single oligodendrocytes coordinate myelin targeting and growth at the same time
• Oligodendrocyte Neurofascin prevents myelination of cell bodies
• Oligodendrocyte Neurofascin promotes myelin sheath growth
• The neuronal binding partner of Neurofascin, Caspr, promotes myelin sheath growth

## Graphical Abstract

**Figure:**
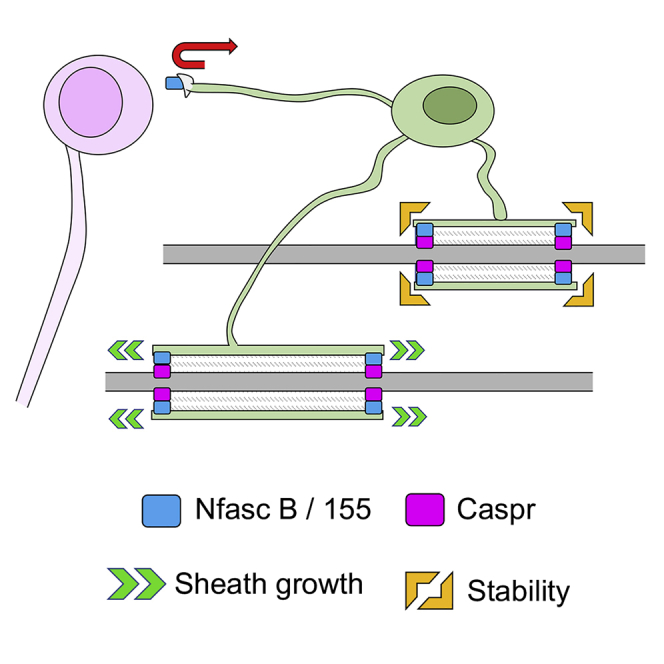

## Teaser

How individual oligodendrocytes coordinate the targeting and growth of myelin is unknown. Klingseisen et al. find that oligodendrocytes employ Neurofascin to prevent myelin mistargeting to cell bodies and also to support the stable growth of sheaths along axons in the CNS of both zebrafish and mice.

## Introduction

Myelination in the central nervous system (CNS), by oligodendrocytes, starts around birth, and continues into adult life, with specific axons and circuits myelinated in stereotyped patterns at distinct times. Myelination speeds up nerve impulse propagation (Seidl, 2014), provides support to axons (Saab and Nave, 2017) and its dynamic regulation, including by neuronal activity, may represent a form of experience-driven nervous system plasticity (Almeida and Lyons, 2017). Although myelination occurs throughout life, the period during which individual oligodendrocytes form and grow their myelin sheaths is, by comparison, very short. Studies in zebrafish and rodents indicate that oligodendrocytes have a period on the order of hours during which they select axons for myelination and initiate myelin sheath growth (Czopka et al., 2013, Watkins et al., 2008). During this time, oligodendrocytes extend dynamic processes that interact with multiple targets, making myelin sheaths on specific axons, while retracting from incorrect targets, including inappropriate axons and cell bodies (Almeida et al., 2018, Baraban et al., 2018, Czopka et al., 2013, Hines et al., 2015, Mensch et al., 2015). Myelin sheath growth continues over a days-long period (Auer et al., 2018, Snaidero et al., 2014), with sheaths remaining stable thereafter (Auer et al., 2018, Hill et al., 2018, Hughes et al., 2018). Although recent studies have provided insight into the dynamics of CNS myelination, the mechanisms by which oligodendrocytes coordinate myelin targeting and growth remain unclear.

Oligodendrocytes can differentiate and enwrap inert axon and cell body shaped structures with myelin in the absence of axonal signals *in vitro* (Bechler et al., 2015, Lee et al., 2012, Redmond et al., 2016). Indeed, this default drive to make myelin can lead to its mistargeting *in vivo*, as evidenced by the mistargeting of myelin to cell bodies when its production outweighs axonal demand (Almeida et al., 2018). However, myelin mistargeting is rare in the healthy nervous system, because myelination is regulated by extrinsic signals. Axonal diameter and neuronal activity can bias myelination toward specific axons (Bechler et al., 2015, Goebbels et al., 2017, Hines et al., 2015, Koudelka et al., 2016, Lee et al., 2012, Mitew et al., 2018, Wake et al., 2015), and inhibitory signals help prevent myelination of incorrect targets (Almeida, 2018, Klingseisen and Lyons, 2017, Redmond et al., 2016). Myelin sheath growth is also influenced by extrinsic signals, as evidenced by stereotyped patterns of myelination along certain axons (Almeida and Lyons, 2017, Ford et al., 2015, Seidl, 2014), and regulation of sheath length by activity (Etxeberria et al., 2016, Hines et al., 2015, Koudelka et al., 2016). In addition, dysregulation of axon-oligodendrocyte adhesion can impair myelin targeting and sheath growth (Elazar et al., 2019a, Elazar et al., 2019b). However, many questions remain as to how oligodendrocytes coordinate myelin targeting and growth *in vivo*. Do individual oligodendrocytes first ensure correct myelin targeting and only subsequently regulate sheath growth, or can the distinct processes of an individual oligodendrocyte regulate sheath targeting and growth in parallel? Do oligodendrocytes employ distinct molecular mechanisms to regulate targeting and growth, or can certain molecules mediate multiple aspects of myelination?

To better understand mechanisms of myelination, we undertook an ENU-mutagenesis-based genetic screen in zebrafish. We identified a mutation in *neurofascin b*, which caused mistargeting of myelin to cell bodies and impaired myelin sheath growth along axons. Neurofascin B encodes an L1 family immunoglobulin cell adhesion molecule required by oligodendrocytes for correct myelin targeting and growth, phenotypes conserved in mice that lack Neurofascin in oligodendrocytes. Live imaging revealed that individual oligodendrocytes can regulate myelin targeting and sheath growth at the same time, in a Neurofascin-dependent manner. We also found that loss of Caspr, the axonal binding partner of oligodendrocyte Neurofascin, impaired sheath growth, likely reflecting their known association in an adhesion complex at the axon-glial interface (Bhat et al., 2001, Einheber et al., 1997, Sherman et al., 2005). *Caspr* disruption did not affect myelin targeting, indicating that Neurofascin regulates myelin targeting and sheath growth through distinct mechanisms.

## Results

### Mutation of Zebrafish Neurofascin B Leads to Mistargeting of CNS Myelin to Cell Bodies

To identify genes that regulate CNS myelination, we carried out an ENU-mutagenesis-based forward genetic screen using zebrafish (STAR Methods). To screen for mutant phenotypes, we used the transgenic reporter Tg(mbp:EGFP-CAAX), in which membrane-localized Green Flourescent Protein (GFP) in myelinating glia allows assessment of myelin morphology (Almeida et al., 2011). We screened zebrafish larvae for disruption to myelination at 5 days post-fertilization (dpf), a time when several circuits are robustly myelinated (Almeida et al., 2011, Koudelka et al., 2016).

**Figure 1:**
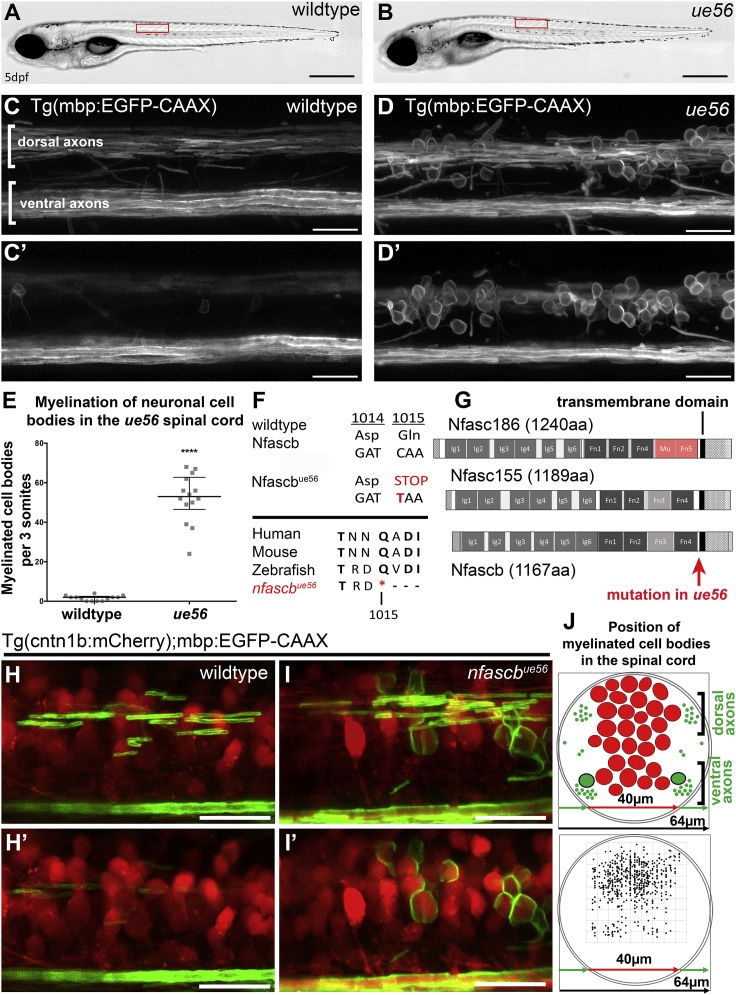
*Neurofascin B* Is Required for Myelin Targeting in the Zebrafish CNS (A and B) Images of wild type (A) and *ue56* mutant (B) larvae at 5 days post-fertilization (dpf), showing normal morphological development of *ue56* mutants. Scale bar, 500 μm. (C–D′) Confocal images of myelin in a wildtype larva (C and C′) and *ue56* mutant (D and D′) at 5dpf, visualized using Tg(mbp:EGFP-CAAX). Lateral views, with anterior to the left. (C) and (D) are maximum intensity projections of z sections taken through the entire spinal cord, with (C′) and (D′) being a projection of a subset of z sections centered closer to the midline in the region where cell bodies are prominent and myelinated in mutants. Scale bars, 20 μm. (E) Quantitation of myelinated cell body number in wildtype and *ue56* mutants at 5dpf in a 3 somite long stretch of spinal cord (wild type median = 2, 25^th^ percentile 0, 75^th^ percentile 2.5, n = 13 animals, *ue56* median = 53, 25^th^ percentile 46.5, 75^th^ percentile 62.75, n = 14 animals, p < 0.0001, t test) (F) Sequence at amino acid positions where the *ue56* mutation generates a stop codon and indication of the conserved region across species. (G) Schematic of predicted domain structure of mouse neurofascin 186, neurofascin 155, and zebrafish neurofascin B, with indication of where the mutation in *ue56* mutants resides. (H–I′) Z projections of confocal images of neurons (red) and myelin (green) in a wildtype animal (H and H′) and *nfascb*^*ue56*^ mutant (I and I′), showing myelin sheaths made on axons (H and I) and myelination of cell bodies in mutants in a region with cell bodies only (H′ versus I′). Scale bars, 20 μm. (J) Schematic cross section of the larval zebrafish spinal cord denoting areas with cell bodies (red) and myelinated axons (green) (Top panel). Dorso-ventral and medio-lateral positions of all myelinated cell bodies (dots) in a 3-somite long stretch of spinal cord of 11 mutants (bottom panel).

One of the mutants identified in our screen, *ue56*, while morphologically normal (Figures 1A and 1B), exhibited a striking phenotype, whereby a large number of cell bodies throughout the CNS were ensheathed by mbp:EGFP-CAAX-labeled membrane (Figures 1C–1E). To identify the mutation responsible for this phenotype, we performed whole-genome sequencing (STAR Methods). This revealed genetic linkage of the mutant phenotype with a region of chromosome 23, in which a C to T transition was identified, predicted to introduce a premature STOP codon in the gene *neurofascin b* (Figures 1F and S1; STAR Methods).

In mammals, splice-variant isoforms of *neurofascin* are generated from a single gene locus. For example, Neurofascin 186 is a neuron-specific isoform that localizes to nodes of Ranvier, where it is essential for ion channel clustering (Sherman et al., 2005, Zonta et al., 2011). Neurofascin 155, in contrast, is a myelinating glial-specific isoform (Collinson et al., 1998, Tait et al., 2000), which forms an adhesion complex with axonal Contactin 1 and Caspr at the paranodal junction (Charles et al., 2002). In contrast to mammals, zebrafish have two Neurofascin-encoding loci, *neurofascin a* and *neurofascin b* (STAR Methods). *neurofascin a* is predicted to encode multiple isoforms, and previous studies have shown that a *neurofascin a* product localizes to nodes of Ranvier (Auer et al., 2018) and is essential for node formation (Voas et al., 2009). However, a gene encoding a zebrafish Neurofascin analogous to the myelinating glial Neurofascin 155 of mammals has remained elusive. Here we find by sequence alignment that *neurofascin b* (*nfascb*) is predicted to encode a protein analogous in domain organization to mammalian Neurofascin 155, in which the extracellular component has six IG domains, four fibronectin domains but lacks the mucin domain characteristic of neuronal isoforms (Figure 1G).

Real-time PCR revealed a complete reduction in *nfascb* mRNA levels in *ue56* mutants (Figure S1B), indicative of nonsense mediated mRNA decay. Therefore, *ue56* is an allele that likely results in a complete loss of Neurofascin B protein. Confirming that disruption of *nfascb* leads to mistargeting of myelin to cell bodies, we found that injection of synthetic mRNA encoding wild-type *nfascb* into *ue56* mutants reduced the number of myelinated cell bodies in mutants (Figures S2A, S2B, and S2E). In addition, knocking down *nfascb* using a morpholino antisense oligonucleotide (STAR Methods), led to an almost identical increase in myelinated cell bodies in the CNS as in *ue56* mutants (Figures S2C, S2D, and S2F). Our observations of myelinated cell bodies in *ue56* mutants, the identification of a STOP codon in *nfascb in ue56* mutants, rescue of the phenotype with wildtype *nfascb* mRNA, and phenocopy by knockdown of *nfascb*, indicate that Neurofascin B is required for correct myelin targeting in the zebrafish CNS. From here we designate the *ue56* mutant *nfascb*^*ue56*^.

### Neurofascin B Prevents Myelination of Cell Bodies in the CNS but Is Dispensable for Targeting of Myelin to Axons

To further assess the role of Neurofascin B in myelin targeting in the zebrafish CNS, we imaged myelinating oligodendrocytes in Tg(cntn1b:mCherry) animals with fluorescently labeled neurons (Figures 1H and 1I′). These analyses confirmed extensive myelination of neuronal cell bodies throughout the spinal cord, with no indication of mistargeting being specific to any particular anterior-posterior region along the neuraxis (Figures 1H and 1I′). When we plotted the medio-lateral and dorso-ventral positions of all myelinated cell bodies in a 3-somite stretch of spinal cord of 11 mutants, they were present at essentially all positions that contained cell bodies, further indicating a general mistargeting of myelin to cell bodies in the absence of Neurofascin B (Figure 1J).

**Figure 2:**
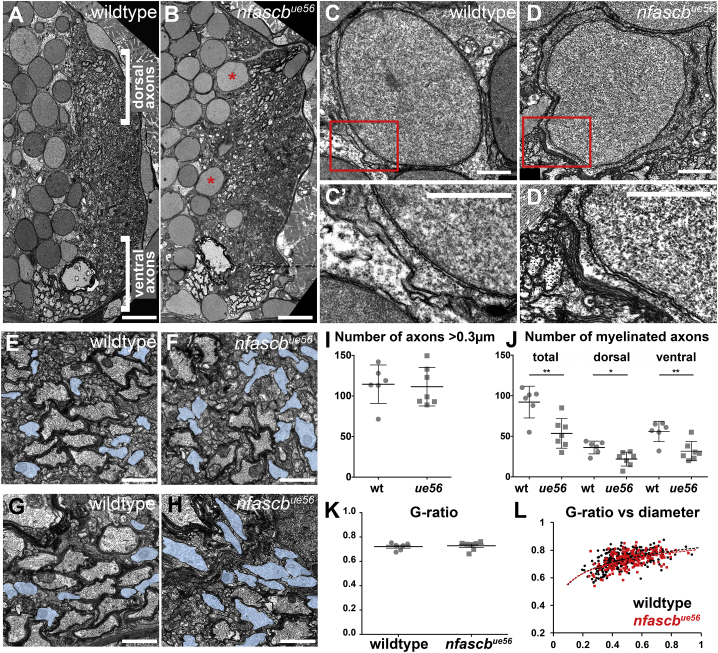
Ultrastructural Analyses of the *nfascb*^*ue56*^ Mutant Spinal Cord (A and B) Transmission electron microscopy (TEM) images of sections through the spinal cord of wildtype (A) and *nfascb*^*ue56*^ mutants (B) at 5 dpf. Dorsal to the top, midline left. Asterisks show myelinated cell bodies. Scale bars, 5 μm. (C–D′) TEM images of cell bodies in wildtype (C and C′) and *ue56* mutants (D and D′) showing enwrapment of a cell body in the mutant with multi-lammellar myelin. Scale bars, 1 μm. (E–H) High magnification views of myelinated axons in the dorsal (E and F) and ventral (G and H) spinal cord of wildtype (E) and mutant (F) animals. Unmyelinated axons with a diameter >0.3 μm highlighted in blue. Scale bars, 0.5 μm. (I) Number of axons with a diameter > 0.3 μm in wildtype and *nfascb*^*ue56*^ mutants (wildtype mean 114.5 ± 23.71 SD, n = 6 animals, *nfascb*^*ue56*^ mutant mean 111.4 ± 23.69 SD, n = 7 animals, p = 0.8201, t test). (J) Total number of myelinated axons in wildtype and *nfascb*^*ue56*^ mutants, and numbers in the dorsal and ventral domains of the spinal cord. (Total: wildtype mean 92.17 ± 19.57 SD and *nfascb*^*ue56*^ mutant mean 53.57 ± 18.20 SD, p = 0.0036, t test. Dorsal: wildtype mean 36.17 ± 7.89 SD and *nfascb*^*ue56*^ mutant mean 21.93 ± 8.60 SD, p = 0.0103, t test. Ventral: wildtype mean 56.00 ± 12.52 SD and *nfascb*^*ue56*^ mutant mean 31.64 ± 11.81 SD, p = 0.0041, t test. All sets wildtype n = 6 animals and *nfascb*^*ue56*^ mutant n = 7 animals). (K) Average g-ratio per animal in wildtype and *nfascb*^*ue56*^ mutants at 5 dpf (wildtype mean 0.72 ± 0.01 SEM, n = 6 animals and *nfascb*^*ue56*^ mutant mean 0.73 ± 0.01 SEM, n = 7 animals, p = 0.7027, t test). (L) G-ratios of all myelinated axons assessed in the dorsal spinal cord of wildtype (black) and *nfascb*^*ue56*^ mutants (red) relative to axon caliber. Each point represents a myelinated axon.

Our analyses of myelination using Tg(mbp:EGFP-CAAX) indicated that despite the extensive myelination of cell bodies, myelin sheaths were made on axons located in the same regions and tracts as controls (Figures 1C and 1D). To better assess axonal myelination, we turned to electron microscopy (EM). This confirmed prominent myelination of cell bodies, many of which were ensheathed with bona fide multilammelar myelin-like membrane (Figures 2A–2D′) and myelination of the same axonal tracts in mutants and controls (Figures 2A and 2B). We have previously shown that in animals with fewer large caliber axons, myelination of cell bodies can ensue (Almeida et al., 2018). Therefore, we wanted to assess whether there was a reduction in large caliber axons in *nfascb*^*ue56*^ mutants that might influence myelin targeting. We saw, however, that the number of large caliber axons was essentially identical in *nfascb*^*ue56*^ mutants and controls (Figures 2E–2I). We did observe, though, that the proportion of such large caliber axons that was myelinated was reduced by about 40% in *nfascb*^*ue56*^ mutants (Figures 2E–2H and 2J), without any evidence of myelination of inappropriately small axons (Figure 2L). Assessment of the thickness of myelin sheaths indicated no difference between wild types and *nfascb*^*ue56*^ mutants (Figures 2 K and 2L).

Given these roles for Neurofascin B in CNS myelination, we also asked how myelination of the peripheral nervous system (PNS) was affected in *nfascb*^*ue56*^ mutants. We assessed myelination of the posterior lateral line nerve (pLLn) by EM, but this revealed no difference in the number of myelinated axons or myelin thickness at 5 dpf (Figure S3). Together our data indicate that Neurofascin B prevents mistargeting of myelin to cell bodies and is required for complete myelination of large caliber axons in the CNS.

### Neurofascin Functions in Oligodendrocytes to Mediate Normal Myelin Targeting

**Figure 3:**
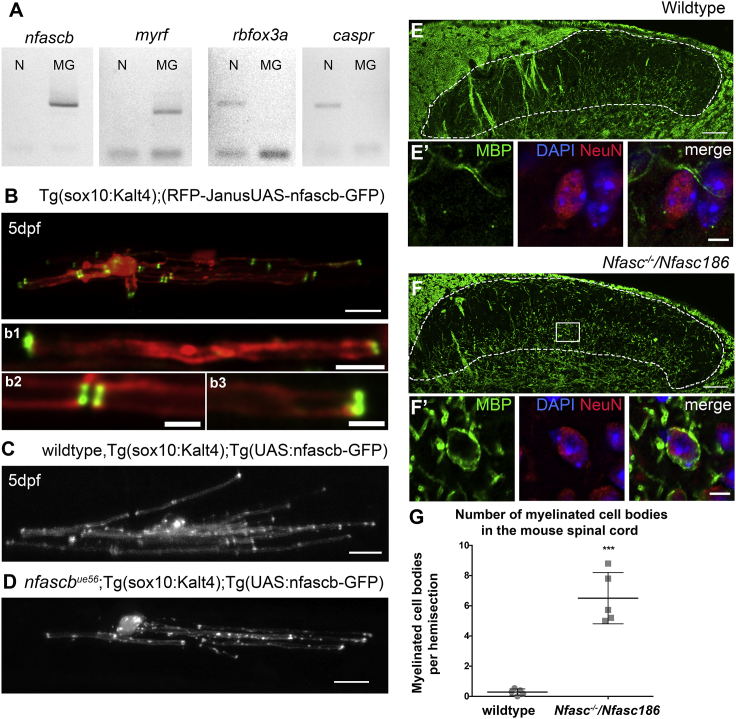
Neurofascin Functions in Oligodendrocytes to Regulate Myelin Targeting (A) Real-time PCR analyses of the expression of *nfascb*, the myelin gene *myrf*, the neuronal genes *rbfox3a* (NeuN) and *caspr* in neuronal (N) and myelinating glial (MG) cells separated using FACS. (B) Confocal images of Nfascb-GFP with cells counterlabelled with mRFP showing concentration of NfascB-GFP at the tips of myelin sheaths (b1-3), where paranodal junctions are localized. Scale bar, 5 μm (top panel) and 2.5 μm (b1-3). (C and D) Confocal images of single oligodendrocytes expressing Nfascb-GFP in wild type (C) and *nfascb*^*ue56*^ mutants (D). Cells expressing wild type NfascB-GFP never myelinate cell bodies, whether they are mutant or wild type. Scale bars, 10 μm. (E–G) Analysis of myelination in mice lacking *Neurofascin* from oligodendrocytes. (E and F) Confocal images of the dorsal horn where assessments for myelination of cell bodies was carried out in control (*Nfasc*^*+/+*^) and *Neurofascin* mutants (*Nfasc*^*−⁄−*^*/Nfasc186*), showing MBP in green, the neuronal marker NeuN in red, and DAPI to indicate nuclei in blue. Insets show single neurons, including an example of a myelinated cell body in the mutant. Scale bar, 50 μm. (G) Quantitation of myelinated cell body number in controls and *Nfasc*^*−⁄−*^*/Nfasc186* mutant mice (wild type mean 0.29 ± 0.21 SD, 10 sections per mouse, n = 5 mice, *Nfasc*^*−⁄−*^*/Nfasc186* mean 6.51 ± 1.70 SD, 10 sections per mouse, n = 5 mice, p < 0.0001, t test).

Since Neurofascin B is analogous to the myelinating glial isoform of mammalian Neurofascin, we next wanted to test whether *nfascb* is expressed in myelinating glia in zebrafish. To do so, we used fluorescence-activated cell sorting (FACS) to collect fluorescently labeled neurons and myelinating glia from transgenic reporters (nbt:dsRed for neurons and mbp:nls-EGFP for myelinating glia) for real-time PCR (STAR Methods). We found that *nfascb* could be amplified from cDNA originating from the myelinating glial cell population, but not the neuronal population (Figure 3A). Previous studies in mammals have shown that the isoform of Neurofascin expressed by myelinating glia is localized to the tips of myelin sheaths at paranodal junctions. To test whether Neurofascin B is similarly localized in zebrafish myelinating oligodendroyctes, we generated a fluorescent fusion protein (Neurofascin B-GFP), which we expressed in oligodendrocytes using sox10 and claudinK gene regulatory sequences (STAR Methods). We observed that Neurofascin B-GFP does indeed localize to the tips of myelin sheaths, further highlighting the similarity of zebrafish Neurofascin B and the myelinating glial Neurofascin of mammals (Figure 3B). To test whether Neurofascin B actually functions in oligodendrocytes to mediate normal myelin targeting, we expressed the Neurofascin B-GFP fusion protein in oligodendrocytes in *nfascb*^*ue56*^ mutant animals. We found that oligodendrocytes that expressed wildtype Neurofascin B-GFP did not myelinate any cell bodies in either control (n = 15) or *nfascb*^*ue56*^ mutants (n = 13), indicating a rescue of the mutant phenotype (Figures 3C and 3D). These data indicate that Neurofascin B is required by oligodendrocytes for correct myelin targeting in the zebrafish CNS.

We next assessed whether myelinating glial Neurofascin is required for myelin targeting in the mammalian CNS. To address this, we generated mice that lacked Neurofascin in myelinating glial cells. Mice in which Neurofascin is conditionally ablated in the oligodendrocyte lineage using *Cre*/*LoxP* approaches die at postnatal day 15–16 (Pillai et al., 2009), an observation that we confirmed (data not shown). Therefore, we generated mice expressing Neurofascin 186, the major neuronal Neurofascin isoform, but lacking the glial Neurofascin 155 isoform. After backcrossing to a C57BL/6 background, this line was interbred with Nfasc^+/−^ mice to generate Nfasc^−⁄−^/Nfasc186 mice as previously described (Zonta et al., 2011). Although these mice display ataxia, they are viable and have a normal life expectancy, presumably due to the continuous production of Neurofascin 186 in neurons throughout life. We assessed whether Nfasc^−⁄−^/Nfasc186 mice exhibited an increase in myelinated cell bodies by examining a region of the dorsal horn of the spinal cord where myelination of cell bodies has been detected in mutants lacking Jam2 (Redmond et al., 2016). We analyzed mutant mice at postnatal day 30 and observed a striking increase in the myelination of cell bodies in Nfasc^−⁄−^/Nfasc186 mutants compared to controls (Figures 3E–3G).

Together our data indicate that oligodendrocyte Neurofascin is required to prevent myelin mistargeting to cell bodies in the CNS of both zebrafish and mice.

### Myelin Sheath Length Is Reduced in the Absence of Oligodendrocyte Neurofascin

**Figure 4:**
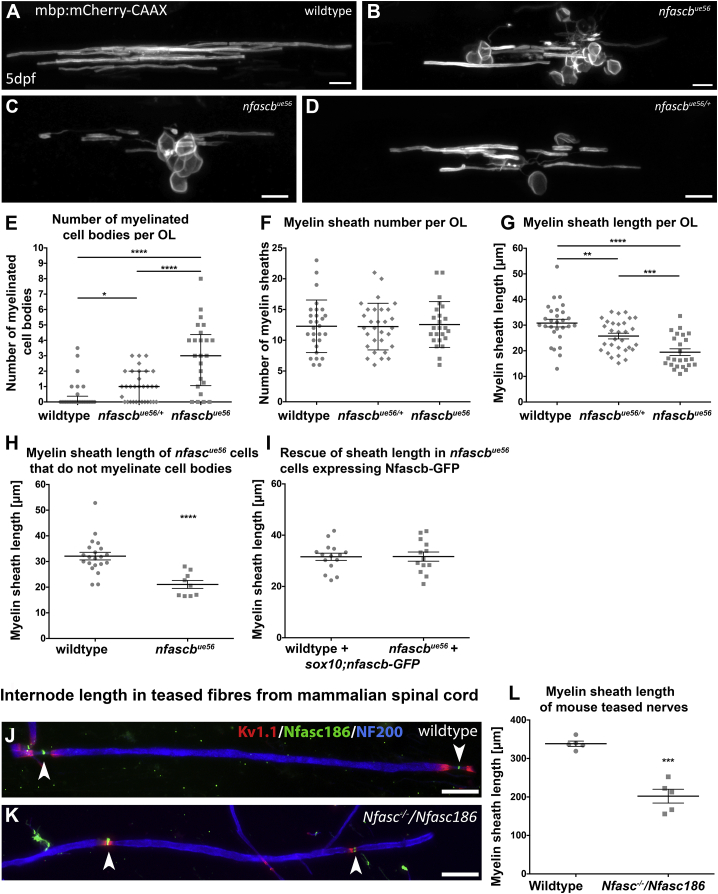
Oligodendrocyte Neurofascin Regulates Both Myelin Targeting and Sheath Length (A–D) Confocal images of single oligodendrocytes labeled mosaically using mbp:mCherry-CAAX in wildtype (A), two *nfascb*^*ue56*^ mutants (B and C), and a heterozygous *nfascb*^*ue56/+*^ animal (D). Scale bars, 10μm. (E–G) Number of myelinated cell bodies (E) and myelin sheaths (F) and the average length of myelin sheaths (G), made by individual oligodendrocytes in wildtype, *nfascb*^*ue56/+*^ hets, and *nfascb*^*ue56*^ mutants. (E) Myelination of cell bodies is increased in both *nfascb*^*ue56/+*^ and *nfascb*^*ue56*^ mutant oligodendrocytes (wildtype median 0.0, 25^th^ percentile = 0.0, 75^th^ percentile = 0.4, n = 28, *nfascb*^*ue56/+*^ median 1.0, 25^th^ percentile = 0, 75^th^ percentile = 2.0, n = 32, *nfascb*^*ue56*^ median 3.0, 25^th^ percentile = 1.1, 75^th^ percentile = 4.4, n = 24; wildtype versus *nfascb*^*ue56/+*^ p = 0.0056, wildtype versus *nfascb*^*ue56*^ p < 0.0001, *nfascb*^*ue56/+*^ versus *nfascb*^*ue56*^ p = 0.0002, all Mann–Whitney test). (F) Number of myelin sheaths per oligodendrocyte is similar in wildtype, *nfascb*^*ue56/+*^ and *nfascb*^*ue56*^ (wildtype mean 12.28 ± 4.27 SD, n = 28, *nfascb*^*ue56/+*^ mean 12.23 ± 3.79 SD, n = 30, *nfascb*^*ue56*^ mean 12.56 ± 3.78 SD, n = 24; wildtype versus *nfascb*^*ue56/+*^. ANOVA = 0.948). (G) Average sheath length per oligodendrocyte in wildtype, *nfascb*^*ue56/+*^, and *nfascb*^*ue56*^ mutants (wildtype mean 30.97 ± 1.50 SEM, n = 28, *nfascb*^*ue56/+*^ mean 25.75 ± 1.12 SEM, n = 30, *nfascb*^*ue56*^ mean 19.46 ± 1.31 SEM, n = 24; wildtype versus *nfascb*^*ue56/+*^ p = 0.0085, wildtype versus *nfascb*^*ue56*^ p < 0.0001, *nfascb*^*ue56/+*^ versus *nfascb*^*ue56*^ p = 0.0005, all t tests). (H) Average sheath length per oligodendrocyte in wildtype and mutant oligodendrocytes with no myelinated cell bodies (wildtype mean 32.11 ± 1.50 SEM, n = 20, *nfascb*^*ue56*^ mean 21.07 ± 1.54 SEM, n = 9; p < 0.0001, Mann–Whitney test). (I) Average sheath length per oligodendrocyte in *nfascb*^*ue56*^ mutants and wildtype siblings injected with wildtype *nfascb*-GFP, which rescues the defect in sheath length (wildtype + *nfascb*-GFP mean 31.58 ± 1.41 SEM, n = 15, *nfascb*^*ue56*^ + *nfascb*-GFP, mean 31.66 ± 1.80 SEM, n = 13, p = 0.9726, t test). (E–I) n in all cases refers to animals. (J and K) Confocal images of teased fiber preparations taken from wild type (K) and *Neurofascin* mutant (J) mice, stained with antibodies that detect Kv1.1 (red), Neurofascin 186 (green) and neurofilament 200 (red). Scale bars, 25 μm. (L) Quantitation of myelin sheath length in *Neurofascin* mutant mice. Lack of Nfasc155 results in a 40% reduction in internode lengths. Wildtype mean 338.4 μm ± 6.88 SEM, n = 5 mice, *Nfasc*^*−⁄−*^*/Nfasc186* mean 202.1 ± 17.94 SEM, n = 5; 50 internodes per mouse, p = 0.0001, t test.

The fact that fewer large caliber axons are myelinated in *nfascb*^*ue56*^ mutants could simply be because oligodendrocytes make so much myelin on cell bodies they do not have enough to target to axons. Alternatively, Neurofascin B might play additional roles in regulating the number and or length of sheaths produced by oligodendrocytes. To address this, we mosaically labeled myelinating oligodendrocytes in zebrafish to assess their morphology (Figure 4; STAR Methods). We saw that individual oligodendrocytes myelinated an average of about 3 cell bodies in *nfascb*^*ue56*^ mutants (Figures 4A–4E). Despite this, the number of myelin sheaths made by individual oligodendrocytes on axons was essentially normal (Figure 4F). These data, together with our reporter and EM analyses, indicate that the targeting of myelin to correct large caliber axons does not require *nfascb*, and that Neurofascin B specifically prevents the mistargeting of myelin to cell bodies in the CNS.

However, our analyses of oligodendrocyte morphology revealed that myelin sheaths were 37% shorter in *nfascb*^*ue56*^ mutants, compared to controls (Figure 4G). Interestingly, myelin sheaths were 35% shorter in the 9/29 *nfascb*^*ue56*^ mutant oligodendrocytes that did not myelinate cell bodies (Figure 4H), indicating that Neurofascin B plays independent roles in myelin targeting and regulation of sheath length. We found no evidence of a role for Neurofascin B in regulating the growth of myelin sheaths in the PNS (Figure S3). Similar to sheath targeting, we found that expression of wildtype Neurofascin B-GFP in oligodendrocytes rescued the reduction in myelin sheath length observed in *nfascb*^*ue56*^ mutants (Figure 4I), indicating that sheath length and targeting are regulated by oligodendrocyte Neurofascin, and that a single *neurofascin b* gene product can ensure both. We also observed an increase in cell body myelination and a reduction in sheath length in *nfascb*^*ue56/+*^ heterozygous animals (Figure 4), indicating that Neurofascin B regulates these distinct aspects of CNS myelination in a dose-dependent manner.

To test whether the role of myelinating oligodendrocyte Neurofascin in regulating sheath length was conserved in mammals, we analyzed internodal distance in mutant mice. To do so, we teased myelinated axons from the ventral spinal cord at postnatal day 30, which revealed a 40% reduction in sheath length (Figures 4J–4L), very similar in extent to that observed in zebrafish, indicating that the role of Neurofascin in controlling myelin sheath length, as well as in regulating myelin targeting, is conserved across vertebrates.

### Neurofascin Independently Regulates the Dynamics of Myelin Targeting and Sheath Growth by Individual Oligodendrocytes

**Figure 5:**
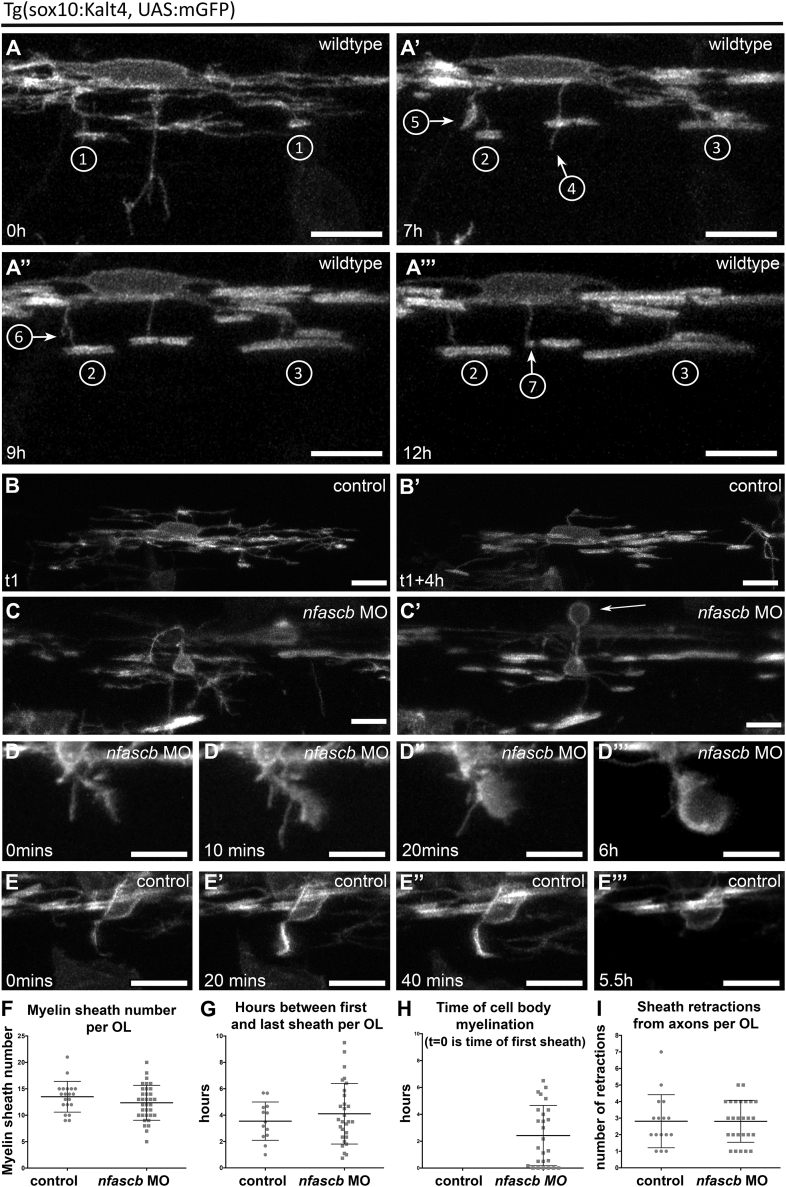
Neurofascin B Regulates Myelin Targeting during the Critical Period of Sheath Formation and Retraction (A–A″′) Single time-point images of an individual oligodendrocyte in a wild-type animal as its myelinating processes exhibit several distinct dynamic activities over time: 1=sheath formation, 2=slow sheath growth, 3=rapid sheath growth, 4=exploratory process retraction, 5=engagement with a cell body, 6=retraction from a cell body, 7=retraction from an axon. Scale bar, 10 μm. (B and B′) Single time-point confocal images of an individual oligodendrocyte in a wildtype animal as it is forming myelin sheaths at the beginning (A) and toward the end (A′) of the critical period. Scale bar, 10 μm. (C and C′) Single time-point confocal images of an individual oligodendrocyte in a *nfascb* MO-injected animal as it is forming myelin sheaths at the beginning (A) and toward the end (A′) of the critical period. Note that a cell body has become myelinated during this time (arrows). Scale bar, 10 μm. (D–D″′) Time series showing myelination of a cell body in a *nfascb* MO-injected animal. Scale bar, 10 μm. (E–E″′) Time series showing a myelinating process being retracted from a cell body in a control. Scale bar, 10 μm. (F) Number of myelin sheaths generated on axons per oligodendrocyte during time-lapse analyses of control and *nfascb* MO-injected animals (control mean 13.50 ± 2.91 SD, n = 20 oligodendrocytes from 13 animals, *nfascb* MO mean 12.35 ± 3.30 SD, n = 34 oligodendrocytes from 17 animals, p = 0.2041, t test). (G) Graph showing the duration between the first formed and the last formed myelin sheath by individual oligodendrocytes in control and *nfascb* MO-injected animals (control mean 3.55 ± 1.45 SD, n = 13 oligodendrocytes from 10 animals, *nfascb* MO mean 4.11 ± 2.30 SD, n = 29 oligodendrocytes from 21 animals, p = 0.4249, t test). (H) Time of cell body myelination during time-lapse of control (none were myelinated) and *nfascb* MO-injected animals. Cell bodies are myelinated by Nfasc B-depleted cells during the same period as axons. (I) Number of myelin sheaths retracted from axons per oligodendrocyte during time-lapse of control and *nfascb* MO-injected animals (control mean 2.81 ± 1.60 SD, n = 16 oligodendrocytes from 13 animals, *nfascb* MO mean 2.81 ± 1.27 SD, n = 26 oligodendrocytes from 17 animals, p = 0.7463, Mann–Whitney test).

Our data show that disruption to oligodendrocyte Neurofascin impairs both myelin targeting and sheath length. To better understand the temporal relationship between targeting and sheath growth, we time-lapse imaged myelination by individual oligodendrocytes in wildtype controls. We imaged membrane-tethered GFP-expressing oligodendrocytes at 2.5 min intervals for 16 h each, starting prior to sheath formation (STAR Methods). We have previously shown that individual oligodendrocytes initiate formation of essentially all of their myelin sheaths on axons within a critical period of 4–6 h (Czopka et al., 2013). Here we wanted to define how the formation of sheaths relates to sheath growth. In all of the oligodendrocytes examined, we observed that during the critical period of sheath formation, distinct cellular processes could engage in entirely different activities at the same time: distinct processes could be forming a new sheath, supporting slow or fast sheath growth along an axon, retracting an exploratory process, or retracting a sheath from a cell body or axon (Figure 5A; Video S1). The fact that these distinct activities can occur at the same time indicates that the mechanisms that control myelin targeting and sheath growth are controlled by distinct localized interactions.

Video S1. Time-Lapse Movie of Oligodendrocyte in Tg(sox10KalTA4, UAS mEGFP) Wild-Type Animal, Related to Figure 5

We next wanted to compare the dynamics of myelin targeting and sheath growth between wildtype and Neurofascin B-depleted animals. We first used the *nfascb*-MO, which phenocopied the *nfascb*^*ue56*^ mutant, because it was not possible to phenotypically identify *nfascb*^*ue56*^ mutants at the start of time-lapsing myelination at 2.5 dpf. We imaged 15 control-injected and 30 *nfascb* MO-injected Tg(sox10:KalTA4, UAS:mEGFP) animals for 15 h at 10 min intervals. We first observed that both the number of sheaths formed and the duration of the critical period of sheath formation were similar in control and *Neurofascin B*-depleted animals (Figures 5B, 5C, 5F, and 5G; Videos S2 and S3), underscoring our observations of normal myelin targeting to axons in *nfascb*^*ue56*^ animals. We next asked when the mistargeting of myelin to cell bodies occurred: was it during the critical period of sheath formation, or later? We found that when oligodendrocytes myelinated cell bodies in *nfascb* morphants they did so during the critical period of sheath formation, concomitant with the formation of sheaths on axons (Figures 5C and 5H; Video S3). We observed that almost all cases of cell body myelination occur by transformation of an exploratory process into a sheet-like protrusion that directly enwraps the cell body (Figure 5D; Video S4). In contrast, exploratory processes that associate with cell bodies in controls almost always subsequently retract from the cell body (Figure 5E; Video S5). In the absence of Neurofascin B, it is possible that oligodendrocytes are simply incapable of retracting myelinating processes from a target. However, the rate of sheath retraction from axons was unaffected in *nfascb* morphants (Figure 5I), indicating that *nfascb*-deficient oligodendrocytes retain the capacity to withdraw processes from targets.

Video S2. Time-Lapse Movie of Oligodendrocyte in Tg(sox10KalTA4, UAS mEGFP) Control-Injected Animal, Related to Figure 5

Video S3. Time-Lapse Movie of Oligodendrocyte in Tg(sox10KalTA4, UAS mEGFP) *nfascb* MO-Injected Animal, Related to Figure 5

Video S4. Time-Lapse Movie of Oligodendrocyte in Tg(sox10KalTA4, UAS mEGFP) *nfascb* MO-Injected Animal, Related to Figure 5

Video S5. Time-Lapse Movie of Oligodendrocyte in Tg(sox10KalTA4, UAS mEGFP) Control-Injected Animal, Related to Figure 5

**Figure 6:**
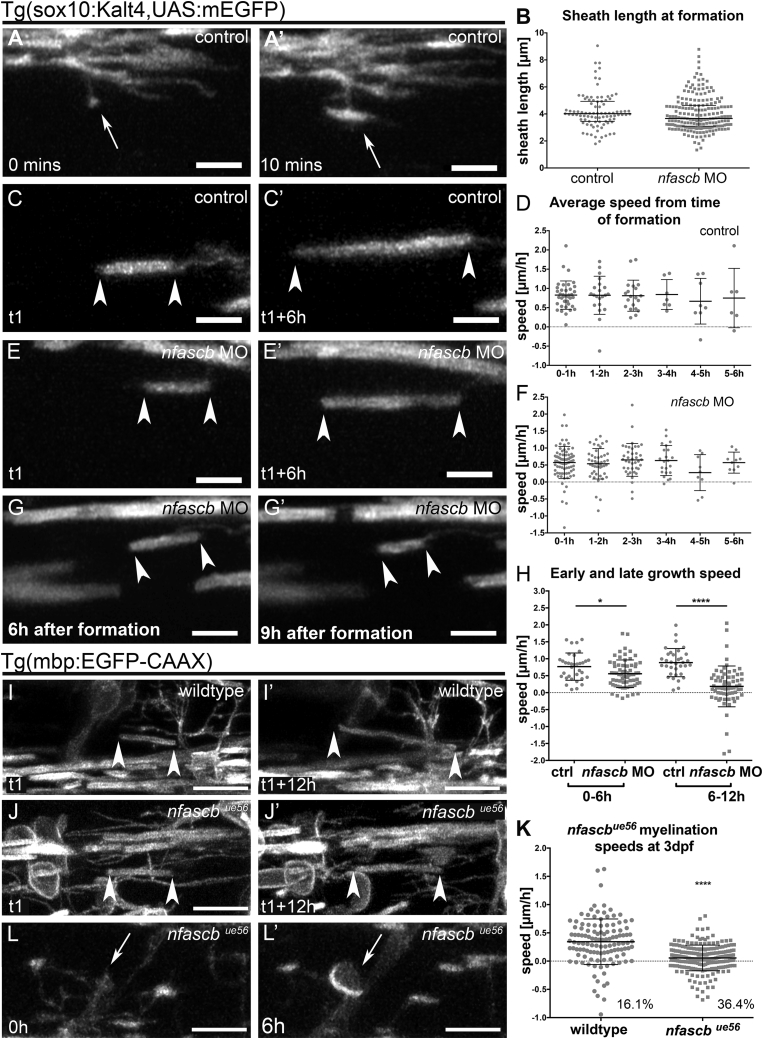
Neurofascin B Regulates Myelin Sheath Elongation and Stability (A and A′) Single time-point confocal images of a myelinating process in a Tg(sox10KalTA4, UAS mEGFP) control that makes contact with a target axon (A) and transitions into a recognizable myelin sheath (A′). Scale bar, 5 μm. (B) Average length of myelin sheaths immediately upon formation in control and *nfascb* MO-injected animals. Control median = 4.02, 25^th^ percentile 3.45, 75^th^ percentile 4.93, n = 91 from 13 animals and *nfascb* MO median = 3.67, 25^th^ percentile 3.10, 75^th^ percentile 4.63, n = 195 sheaths from 21 animals, p = 0.0611, Mann–Whitney test. (C and C′) Confocal images of a myelin sheath elongating over time during the period of sheath formation in a control animal. Arrowheads in all panels denote ends of myelin sheaths. Scale bar, 5 μm. (D) Average speed of individual sheath elongation in controls over a 12-h period, starting from the hour of their formation during the critical period. Each point represents a single sheath, imaged across 13 animals. No significant difference in speed of growth based on time of sheath formation, p = 0.8933, Kruskal-Wallis test. (E and E′) Single time-point confocal images of a myelin sheath elongating over time during the critical period of sheath formation in an *nfascb* MO-injected animal. Scale bar, 5 μm. (F) Average speed of sheath elongation in *nfascb* MO-injected animal over a 12-h period, starting from the hour of their formation during the critical period. Each point represents a single sheath, imaged across 21 animals. No significant difference in speed of growth based on time of sheath formation, p = 0.6301, Kruskal–Wallis test. (G and G′) Single time-point confocal images of a myelin sheath shrinking over time after the critical period in a *nfascb* MO-injected animal. Scale bar, 5 μm. (H) Speed of elongation of individual myelin sheaths in control and *nfascb* MO-injected animals for the first 6 h after their formation and the following 6 h. (control n = 34 sheaths in 8 animals, *nfascb* MO n = 66 sheaths in 13 animals: 0–6 h: control mean speed 0.77 ± 0.40 SD, *nfascb* MO mean 0.56 ± 0.41 SD, p = 0.0133, Mann–Whitney-test. 6–12 h: control mean speed 0.89 ± 0.42 SD, *nfascb* MO mean 0.185 ± 0.60 SD, p < 0.0001, Mann–Whitney test. Note the large number of sheaths shrinking in *nfascb* MO-injected animals between 6–12 h after sheath formation. (I and I′) Confocal images of a myelin sheath elongating in a wildtype animal. Scale bar, 10 μm. (J and J′) Confocal images of a myelin sheath elongating in an *nfascb*^*ue56*^ mutant. Scale bar, 10 μm. (K) Speed of myelin sheath elongation in Tg(mbp:EGFP-CAAX) wildtype and *nfascb*^*^ue56^*^ mutants (control mean speed 0.34 ± 0.40 SD, n = 118 from 6 animals, *nfascb*^*ue56*^ mutant mean 0.06 ± 0.23 SD, n = 215 from 9 animals, p < 0.0001, Mann–Whitney test. 16.1% in controls and 36.4% in *nfascb*^*ue56*^ represent shrinking myelin sheaths. (L) Cell body myelination in *nfascb*^*ue56*^ mutants. Scale bar, 10 μm.

We next wanted to understand the reduction in sheath length in *nfascb*-disrupted animals. We first assessed the initial appearance of myelin sheaths made on axons. We found that immediately upon their formation, myelin sheaths were on average 4 μm in length in both wildtype and *nfascb* MO-injected animals (Figures 6A and 6B), further indicating that the initial formation of myelin sheaths is independent of Neurofascin B. We next wanted to determine whether Neurofascin B is required to ensure normal myelin sheath growth during a specific phase of sheath elongation, continuously during elongation, or simply to maintain stable myelin sheaths once elongated. We first plotted the rate of sheath elongation relative to the time of their formation by an individual oligodendrocyte. We saw that sheaths grew at similar speeds whether they were the first or last made by an oligodendrocyte. Although the speed of sheath growth is variable (Figures 6D and 6F), the average speed of growth is reduced in *nfascb*-morphants from soon after their formation (Figures 6C–6F; Videos S6 and S7), a phenotype that becomes more pronounced over time (Figures 6G and 6H). We found that this reduction in the average speed of sheath growth reflects a reduction in the speed of positive elongation, an increase in the number of sheaths that fail to elongate and an increase in those that shrink over time (Figure 6H; Video S8). These results suggest that *nfascb* is required to support the normal growth of myelin sheaths along axons soon after their formation. To corroborate our findings in *nfascb*-morphants and examine myelination at a slightly later stage, we time-lapse imaged Tg(mbp:EGFP-CAAX) wildtypes and *nfascb*^*ue56*^ mutants from 3.5 dpf. We observed myelination of cell bodies in mutants (Figure 6L; Video S11), that the speed of myelin sheath growth along axons remained reduced, and that a large proportion of myelin sheaths shrank over time in *nfascb*^*ue56*^ mutants (Figures 6I–6K; Videos S9 and S10).

Video S6. Time-Lapse Movie of a Myelin Sheath Elongating Quickly in a Tg(sox10KalTA4, UAS mEGFP) Control-Injected Animal, Related to Figure 6

Video S7. Time-Lapse Movie of a Myelin Sheath Elongating Slowly in a Tg(sox10KalTA4, UAS mEGFP) *nfascb* MO-Injected Animal, Related to Figure 6

Video S8. Time-Lapse Movie of a Myelin Sheath Shrinking in a Tg(sox10KalTA4, UAS mEGFP) *nfascb* MO-Injected Animal, Related to Figure 6

Video S9. Time-Lapse Movie of a Myelin Sheath Growing in a Tg(sox10KalTA4, UAS mEGFP) Wild-Type Animal, Related to Figure 6

Video S10. Time-Lapse Movie of a Myelin Sheath Growing in a Tg(sox10KalTA4, UAS mEGFP) *nfascb*^*ue56*^ Animal, Related to Figure 6

Video S11. Time-Lapse Movie of Myelination of a Cell Body in a Tg(sox10KalTA4, UAS mEGFP) *nfascb*^*ue56*^ Animal, Related to Figure 6

Together our data indicate that oligodendrocyte Neurofascin mediates two distinct roles during myelination: (1) preventing the myelination of cell bodies, and (2) promoting the stable elongation of sheaths along axons.

### Caspr Regulates Sheath Elongation but Not Myelin Targeting

The fact that oligodendrocyte Neurofascin prevents myelination of cell bodies and promotes the growth of sheaths along axons begs the question as to what drives these distinct aspects of myelination. One possibility is that interactions with different molecules on neuronal cell bodies versus axons regulate the outcome. Along the axon, it is well established that myelinating glial Neurofascin 155 interacts with the cell adhesion molecules Caspr and Contactin 1 (Bhat et al., 2001, Boyle et al., 2001, Çolakoğlu et al., 2014). Although previous studies have indicated that disruption to Contactin 1 leads to severe disruption to axonal growth and myelination in the CNS (Çolakoğlu et al., 2014), loss of Caspr function may more specifically dysregulate axon-glial adhesion (Brivio et al., 2017).

**Figure 7:**
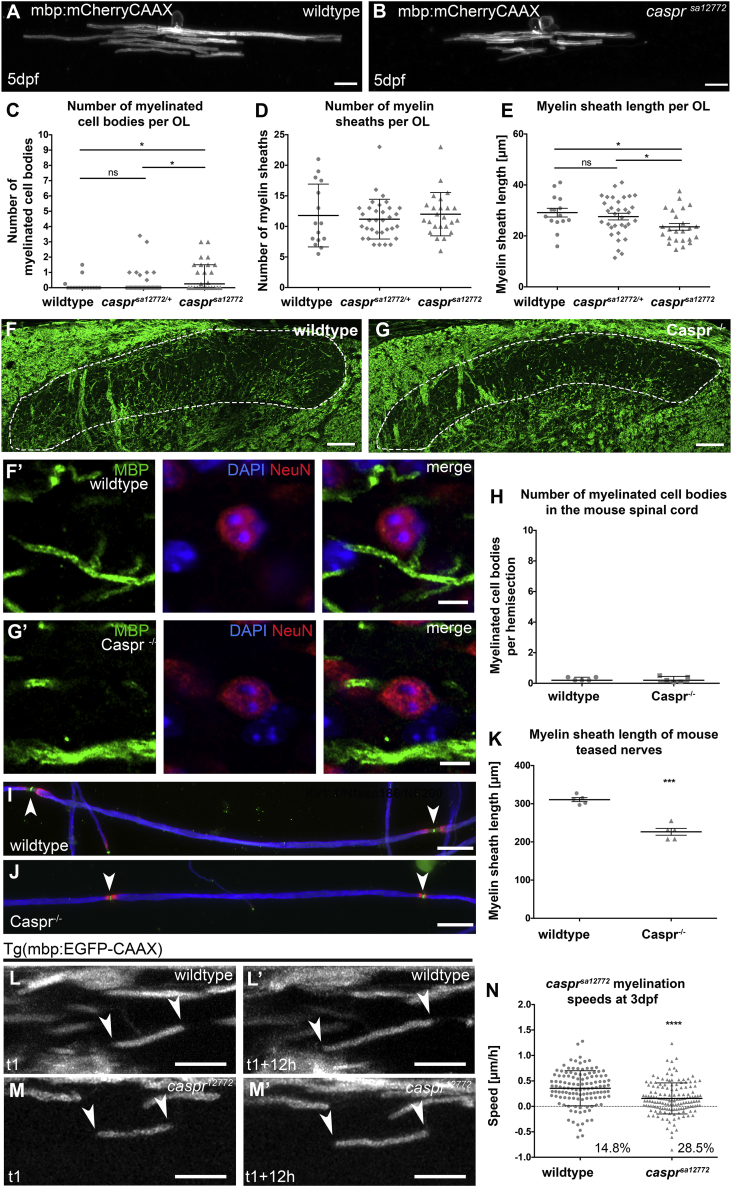
Caspr Regulates Myelin Sheath Elongation and Stability, but Not Targeting (A and B) Confocal images of single oligodendrocytes labeled mosaically using mbp:mCherry-CAAX in wildtype (A) and *caspr*^*sa12772*^ mutants at 5 dpf. Scale bar, 10 μm. (C–E) Number of myelinated cell bodies (C), number of myelin sheaths (D), and the length of myelin sheaths (D), made by individual oligodendrocytes in wildtype, het, and *caspr*^*sa12772*^ mutants. (C) Myelination of cell bodies is increased slightly in *caspr*^*sa12772*^ mutant oligodendrocytes (wildtype median 0.0, 25^th^ percentile 0.0, 75^th^ percentile 0.0, n = 15 animals; *caspr*^*sa12772/+*^ median 0.0, 25^th^ percentile 0.0, 75^th^ percentile 0.0, n = 35, *caspr*^*sa12772*^ median 0.25, 25^th^ percentile 0.0, 75^th^ percentile 1.5 , n = 25 animals; wildtype versus *caspr*^*sa12772/+*^ p = 0.8056, wildtype versus *caspr*^*sa12772*^ p = 0.0305, *caspr*^*sa12772/+*^ versus *caspr*^*sa12772*^ p = 0.0156, all Mann–Whitney test. (D) Number of myelin sheaths per oligodendrocytes is similar in wildtype, *caspr*^*sa12772/+*^ and *caspr*^*sa12772*^ (wildtype mean 11.78 ± 5.14 SD, n = 15 animals, *caspr*^*sa12772/+*^ mean 11.19 ± 3.26 SD, n = 33 animals, *caspr*^*sa12772*^ mean 12.00 ± 3.54 SD, n = 24; ANOVA = 0.712). (E) Myelin sheath length is reduced in *caspr*^*sa12772*^ mutants (wildtype mean 29.14 ± 1.71 SEM, n = 15, *caspr*^*sa12772/+*^ mean 27.58 ± 1.31 SEM, n = 33 animals, *caspr*^*sa12772*^ mean 23.55 ± 1.30 SEM, n = 24 animals; wildtype versus *caspr*^*sa12772/+*^ p = 0.4954, wildtype versus *caspr*^*sa12772*^ p = 0.0124, *caspr*^*sa12772/+*^ versus *caspr*^*sa12772*^ p = 0.0374, all t test). (F and G) Confocal images of the dorsal horn of the spinal cord in wildtype (F) and Caspr mutant (G) mice, indicating the region indicated by dashed lines wherein myelinated cell bodies were searched for. Scale bar, 50 μm. (F′ and G′) Higher magnification views of regions within the dorsal horn of wild type (F′) and *Caspr* mutant (G′) animals stained with antibodies that recognize myelin basic protein (green), NeuN (red), and DAPI (blue). Scale bar, 5 μm. (H) Graph showing that essentially no myelinated cell bodies were observed in wild type or *Caspr* mutant animals. Wild type mean 0.2800 ± 0.049 SEM, n = 5 (10 sections per mouse), Caspr^−⁄−^ 0.257 ± 0.083 SEM, n = 5 (10 sections per mouse), p = 0.8147, t test. (I and J) Confocal images of teased fiber preparations taken from wild type (I) and Caspr mutant (J) mice, stained with antibodies that detect Kv1.1 (red), Neurofascin 186 (green), and neurofilament 200 (red). Scale bars, 25 μm. (K) Quantitation of myelin sheath length in Caspr mutant mice. Caspr internode lengths are reduced compared to wild type; wild type mean 310.7 ± 5.32 SEM, n = 5, Caspr 226.5 ± 8.96 SEM, n = 5 animals, 50 internodes per mouse, p < 0.0001, t test. (L and L″) Confocal images of a myelin sheath elongating in a wildtype Tg(mbp:EGFP-CAAX) animal. Scale bar, 10 μm. (M and M′) Confocal images of a myelin sheath elongating in a *caspr*^*sa12772*^ mutant. Scale bar, 10 μm. (N) Speed of myelin sheath elongation in wildtype and *caspr*^*sa12772*^ mutants: wildtype mean 0.36 ± 0.35 SD, n = 122 sheaths from 6 animals, *caspr*^*sa12772*^ mean 0.16 ± 0.31 SD, n = 151 sheaths from 7 animals, p < 0.0001, Mann–Whitney test. 14.8% in controls and 28.5% in *nfascb*^*ue56*^ represent shrinking myelin sheaths.

Therefore, we sought to test whether Caspr might also regulate myelin targeting and growth. We first analyzed myelination in a zebrafish mutant generated through the zebrafish mutation project (Kettleborough et al., 2013), in which a T to A mutation (*sa12772*) induces a STOP codon at amino acid 200 of 1,310 of Caspr (STAR Methods). Similar to *nfascb*^*ue56*^ mutants, the number of myelin sheaths made by individual oligodendrocytes was unaffected in *caspr*^*sa12772*^ mutants, indicating no requirement for Caspr in targeting myelin to axons in the CNS (Figures 7A, 7B, and 7D). Although we did observe a modest increase in the number of myelinated cell bodies made by individual oligodendrocytes in *caspr*^*sa12772*^ mutants (Figure 7C), this was 10-fold lower in magnitude to the phenotype observed in *nfascb*^*ue56*^ mutants (Compare median myelinated cell number per OL in Figures 4E and 7C). Further arguing against a specific role for Caspr in myelin targeting, we observed no evidence of myelin mistargeting to cell bodies in Caspr mutant mice (Figures 7F–7H), which we analyzed at postnatal day 23 in the same region of the dorsal spinal cord in which we observed myelin mistargeting to cell bodies in Neurofascin mutant mice. Together our data from both zebrafish and mice indicate that Caspr does not play a major role in regulating myelin targeting in the CNS. This suggests that Neurofascin mediates its role in preventing myelin targeting to cell bodies in a Caspr-independent manner, the basis of which remains to be determined.

Our analyses of myelination by individual oligodendrocytes, did however, indicate a very clear reduction in myelin sheath length in zebrafish *caspr*^*sa12772*^ mutants (Figure 7E). Similarly, analysis of internodal length in Caspr mutant mice teased nerves also exhibited a reduction in sheath length (Figures 7I–7K), indicating conservation in Caspr function between zebrafish and mice, as we observed for oligodendrocyte Neurofascin. To test to what extent Caspr deficiency phenocopied the defects in sheath elongation and stability observed in *nfascb*-deficient animals, we carried out time-lapse imaging of myelination in *caspr*^*sa12772*^ mutant zebrafish. As observed in *nfascb* morphants and mutants, the speed of sheath elongation was significantly reduced in *caspr*^*sa12772*^ mutants (Figures 7L–7N), and the proportion of myelin sheaths that shrank over time increased (Figure 7N). Together these data indicate that Caspr supports myelin sheath elongation along axons in a similar manner to Neurofascin B, likely due to their association in an adhesion complex at the axon-glial interface.

## Discussion

To identify mechanisms of myelination we carried out a genetic screen in zebrafish, which, together with analyses in mice, identified roles for oligodendrocyte Neurofascin in preventing the mistargeting of myelin to cell bodies and supporting the growth of myelin sheaths, and for Caspr in promoting sheath growth. Our time-lapse analysis showed that the distinct myelinating processes of single oligodendrocytes can engage in myelin targeting and growth at the same time and that Neurofascin mediates its roles in myelination independently of one another. Oligodendrocytes can myelinate cell body shaped plastic beads (Redmond et al., 2016) and generate myelin sheaths along synthetic microfibers, (Bechler et al., 2015), with such myelination taking place in the absence of specific molecular interactions of myelinating processes and their targets. Myelination in the absence of oligodendrocyte Neurofascin may resemble such default myelination, with stochastic targeting of myelin to biophysically permissive substrates and unstable sheath growth along axons. Therefore, Neurofascin-dependent interactions between myelinating processes and targets represent key mediators of myelin targeting and growth *in vivo*. We now need to ask: how can Neurofascin mediate such distinct aspects of myelination within the same cell at the same time?

At present, it is unclear how oligodendrocyte Neurofascin prevents myelination of cell bodies. Our time-lapse analyses indicate that Neurofascin mutant oligodendrocytes retain the capacity to retract myelinating processes from axons during myelination. Therefore, the simplest model is that a signal localized to cell bodies interacts with Neurofascin on myelinating processes and triggers retraction from the cell body. The junctional adhesion molecule Jam2 is an inhibitory signal that can prevent myelination of cell bodies (Redmond et al., 2016). Although interaction between neuronal Jam2 and oligodendrocyte Neurofascin cannot be ruled out, the extracellular domains of junctional adhesion molecules primarily bind other JAM molecules or integrins (Bazzoni, 2003), and the myelination of cell bodies in Jam2 mutants is restricted to a specific neuronal subtype (Redmond et al., 2016), unlike the broad myelination of cell bodies observed in Neurofascin mutants. To date, the key known binding partners of oligodendrocyte Neurofascin155 are Caspr and Contactin 1. Our study indicates that Caspr does not play a prominent role in preventing myelin targeting to cell bodies. Although Caspr and Contactin 1 are co-dependent at the paranodal junction, it remains possible that Contactin 1 could serve as an inhibitory cue that prevents myelination of cell bodies. There is evidence that Caspr can prevent direct binding of Contactin 1 to Neurofascin 155 (Gollan et al., 2003), and Caspr is not normally localized to neuronal cell bodies (Boyle et al., 2001, Redmond et al., 2016). Therefore, a Caspr-independent direct binding of Contactin 1 to Neurofascin could inhibit myelination of cell bodies. Testing this will require extensive cell-type specific loss and gain of Contactin 1 function approaches, because Contactin 1 mutant mice exhibit relatively severe disruption to both axonal development and myelination (Çolakoğlu et al., 2014). In addition, other unknown factors might bind to or regulate oligodendrocyte Neurofascin. Neurofascin 155 is subject to post-translational modification, including cleavage by metalloproteases (Maier et al., 2006), indicating that its context dependent processing might contribute to its distinct roles.

Insight into how Neurofascin might affect sheath growth comes from analyses of its role at the axon-glial paranodal junction. Mutant mice with complete loss of either Neurofascin (Zonta et al., 2008) or Caspr (Brivio et al., 2017) have longer gaps between adjacent myelin sheaths at nodes of Ranvier. However, expression of Neurofascin 155 in myelinating glia or Caspr in neurons is sufficient to restore formation of the paranodal complex and to rescue the increase in nodal gap length in each respective mutant. Our observations of reduced sheath length in both *neurofascin* and *caspr* mutant fish and mice suggest that the longer nodal gaps seen in corresponding mutants might reflect slower myelin sheath elongation. However, the rescue of nodal gap length brought about by expression of Neurofascin 155 in myelinating glia does not require its intracellular domain (Zonta et al., 2008). In contrast, closing of the nodal gap does require the intracellular domain of Caspr (Brivio et al., 2017). These data indicate that closing of the gap mediated by the axon-glial adhesion complex is driven by the axon and not by the myelinating process. Testing whether myelin sheath growth might also be driven by a Caspr-Neurofascin interaction in an inside-out, axon to myelin, manner would require extensive investigations of sheath growth in animals with specific disruption to the intracellular signaling of oligodendrocyte Neurofascin and neuronal Caspr. Alternatively, the deficits we observe in myelin sheath length in Neurofascin and Caspr mutants may simply reflect their formation of an adhesion complex that supports sheath growth. Indeed, dysregulation of axon-myelin adhesion has recently been shown to impair myelination in the CNS (Elazar et al., 2019b).

In summary, oligodendrocyte Neurofascin regulates both myelin targeting and growth at the same time in individual cells. The functional consequences of cell body myelination and impaired growth need to be disentangled, which will require in depth investigation and comparison of mutants with disruption to oligodendrocyte and neuronal Caspr. This will be important, not only to understand fundamental mechanisms, but also to gain insight into the increasing number of human diseases in which disruption to oligodendrocyte Neurofascin or axonal Caspr are evident (Efthymiou et al., 2019, Hinman et al., 2006, Howell et al., 2006, Low et al., 2018, Smigiel et al., 2018). The mechanistic dissection of how oligodendrocyte Neurofascin, as a single cell adhesion molecule, can regulate two distinct and essential aspects of myelination in the same cell at the same time also represents an important challenge for the future.

## STAR★Methods

### Key Resources Table

**Table:** 

REAGENT or RESOURCE	SOURCE	IDENTIFIER
**Antibodies**		

Mouse Anti-NeuN IgG1 Clone A60, 1:500	Millipore	Cat#MAB377, RRID:AB_2298772
Mouse Anti-Caspr IgM, 1:50	Dr. Matthew Rasband	N/A
Mouse Anti-NF200, 1:20000	Sigma	Cat#N0142, RRID:AB_477257
Mouse Anti- Kv 1.1 IgG2b, 1:200	Neuromab	Cat#75–105, RRID:AB_2128566
Rabbit Anti-MBP Pep-7, 1:1000	Dr. Peter Brophy	N/A
Rabbit Anti-MNF2 (Anti-Nfasc186), 1:1000	Dr. Peter Brophy	N/A
Goat Anti-Mouse AlexaFlour 488, 1:1000	Thermofisher	Cat#A-21141, RRID:AB_2535778
Goat Anti-Mouse AlexaFlour 594, 1:1000	Thermofisher	Cat#A-21125, RRID:AB_2535767
Goat Anti-Mouse AlexaFlour 647, 1:1000	Thermofisher	Cat#A-21240, RRID:AB_2535809
Goat Anti-Mouse FITC, 1:100	Southern Biotec	Cat# 1021-02, RRID:AB_2794237
Goat Anti-Rabbit AlexaFlour 488, 1:1000	Molecular Probes	Cat# A-11008, RRID:AB_143165
Donkey Anti-Rabbit AlexaFlour 594, 1:1000	Jackson ImmunoResearch	Cat#111-585-14,RRID:AB_2307325

**Experimental Models: Organisms/Strains**		

Zebrafish: Tg(mbp:EGFP-CAAX)	(Almeida et al., 2011)	ZFIN: ZDB-ALT-120103-2
Zebrafish: Tg(mbp:nlsEGFP)	(Karttunen et al., 2017)	
Zebrafish: Tg(NBT:DsRed, Synon. Tg(Xla.Tubb:DsRed), zf148Tg	(Peri and Nüsslein-Volhard, 2008)	ZDB-ALT-081027-2
Tg(cntn1b:mCherry)	(Czopka et al., 2013)	ZDB-TGCONSTRUCT-140610-5
Zebrafish: Tg(sox10:KalTA4)	(Almeida and Lyons, 2015)	N/A
Tg(cldnk:Gal4)	(Münzel et al., 2012)	ZDB-TGCONSTRUCT-120207-1
Zebrafish: Tg(UAS:Mem-GFP)	(Kwan et al., 2007)	N/A
Zebrafish: *caspr*^*sa12772*^	(Kettleborough et al., 2013)	ZDB-ALT-130530-311
Zebrafish: n*fascb*^*^ue56^*^	this paper	N/A
Mouse: Nfasc-/-/Nfasc186	(Zonta et al., 2011)	N/A
Mouse: Caspr-/-	(Gollan et al., 2003)	N/A

**Oligonucleotides**		

Morpholino: ATG-nfascb, TGACAGGAATCCTCCAACACTTCAT	Gene Tools	N/A
Morpholino: Human Beta-Globin, Standard Negative Control, CCTCTTACCTCAGTTACAATTTATA	Gene Tools	N/A
nfascb_ORF_F	ACAAAATTACACCAAAACGCTGT	N/A
nfascb_ORF_R	AGCCAGTCTGACAGATTAAATGGA	N/A
nfascb_geno143_F	TTGCATGCCTGAGCAGAATA	N/A
nfascb_geno143_R	TACTTTCCTCCTCGGCTCCT	N/A
Caspr_geno803_F	TTTCACACCAACAACATGGAA	N/A
Caspr_geno803_R	GGGAAGGGTGGATGGAATAA	N/A
Nfascb_179-975_RT_F	TCCAGTCTTCACATGGACGC	N/A
Nfascb_179-975_RT_R	CGGAACCGATTTGACCCTGA	N/A
mbpX3_RT_F	AGAAAGGGAAAGAGACCCCAC	N/A
mbpX3_RT_R	GATCGGCTTTCTCCCAGGTT	N/A
nfasc_FACS-RT_F	GGCCCTCCTAAACCAGACAC	N/A
nfasc_FACS-RT_R	ATGGGTTTGAAGCGTTGCAC	N/A
rbfox3a_FACS-RT_F	AGGGACCAGCAGCTTAACAC	N/A
rbfox3a_FACS-RT_R	GCGACTGTAACCTCCTCTGT	N/A
myrf_FACS-RT_F	AATCGTTCTGGGGAACTCGG	N/A
myrf_FACS-RT_R	GATCGTTAGCTTGCTGGGGT	N/A
Caspr_FACS-RT_ F	TTTCCGGAAGAACCGTCTGG	N/A
Caspr_FACS-RT_ R	CTTCCCCCTTGTAGCCTGTG	N/A
attB1_nfascb_F	GGGGACAAGTTTGTACAAAAA AGCAGGCTGCCACCATGAAGTG TTGGAGGATTCCTGTC	N/A
Nfascb_noStop18_F	CTCGCCCTTGCTCACCATAGCAAA AGAGTAGATGGCCACAGGAGATGT GGGCTCAGAG	N/A
18_EGFP_F	GCCATCTACTCTTTTGCTATGGTGA GCAAGGGCGAGGAGCTGTTCACC GGGGTGG	N/A
attB2R_EGFP_R	GGGGACCACTTTGTACAAGAAAGCTG GGTTTACTTGTACAGCTCGTCCATGCC	N/A
attB4_nfascb_F	GGGGACAACTTTGTATAGAAAAGTTGAA AAAACCTCCCACACCTCCCCC	N/A
attB1R_nfascb_R	GGGGACTGCTTTTTTGTACAAACTTGGC CACCATGAAGTGTTGGAGGATTCCTGTC	N/A

**Recombinant DNA**		

pTol2- UAS:nfascb-EGFP-pA	This paper	N/A
pTol2- nfascB-EGFP-UAS-mCherry	This paper	N/A
Tol2 Kit	(Kwan et al., 2007)	http://tol2kit.genetics.utah.edu/index.php/ Main_Page

**Software and Algorithms**		

Zen, Zen Blue	Zeiss	RRID:SCR_013672
Fiji	(Schindelin et al., 2012)	RRID:SCR_002285
GraphPad Prism	GraphPad software	RRID:SCR_015807
Adobe Photoshop	Adobe	RRID:SCR_014199

### Lead Contact and Materials Availability

Further information and requests for resources and reagents should be directed to and will be fulfilled by the Lead Contact, David Lyons (david.lyons@ed.ac.uk).

### Experimental Model and Subject Details

#### Zebrafish Lines

All zebrafish were maintained under standard conditions in the Queen’s Medical Research Institute CBS Aquatics facility at the University of Edinburgh. All mouse work conformed to UK legislation (Scientific Procedures Act of 1986), and to University of Edinburgh Ethical Review Committee policy. Studies were carried out with approval from the UK Home Office and according to its regulations, under project licenses 60/8436 and 70/8436. Adult animals were kept in a 14 hours light and 10 hours dark cycle. Embryos were kept at 28.5°C in 10mM HEPES-buffered E3 Embryo medium or conditioned aquarium water with methylene blue.

Throughout the text and in figures, ‘Tg’ denotes a stable, germline inserted transgenic line.

#### The Following Existing Mutant and Transgenic Lines Were Used

Tg(mbp:EGFP-CAAX) (Almeida et al., 2011) Tg(mbp:nlsEGFP) (Karttunen et al., 2017) Tg(Xla.Tubb:DsRed) referred to as Tg(NBT:DsRed) (Peri and Nüsslein-Volhard, 2008); Tg(sox10:KaltA4)(Almeida and Lyons, 2015), Tg(UAS:mem-GFP) (Kwan et al., 2007), *caspr*^*sa12772*^(Kettleborough et al., 2013): note the *caspr* gene is also referred to as *cntnap1* or *caspr1*. The *nfascb*^*ue56*^ mutant was identified in the genetic screen described in this manuscript.

#### ENU Mutagenesis and Screen

10 adult AB males were mutagenized with 3.5mM ENU for 1 hr per week over three consecutive weeks. Assessment of mutagenesis efficiency was made by crossing with carriers of a mutation, *sox10*^*cls*^ (Kelsh et al., 1996), which disrupts pigment formation. Well-mutagenised males were crossed with AB females to generate the F1 generation. F1 individuals were bred with Tg(mbp:GFP-CAAX) animals to introduce the myelin reporter into the mutagenized stocks and generate individual F2 families. We generated 212 F2 families, and screened 946 clutches from these families for disruption to mbp:EGFP-CAAX expression at 5 dpf. See Kegel et al. (2019)Kegel et al., 2019 for extensive details on mutagenesis protocol, assessment of mutagenesis efficiency and breeding scheme prior to screen.

#### Mapping-By-Sequencing

Following an outcross to WIK, pooled DNA from 100 mutant recombinants and pooled DNA from 150 sibling recombinants was sequenced separately on an Illumina HiSeq4000 (Edinburgh Genomics) to a coverage of ∼18X. We processed this data through a slightly modified version of the Variant Discovery Mapping (VDM) CloudMap pipeline (Minevich et al., 2012), on an in-house Galaxy server using the Zv9/danRer7 genome and annotation. For both the VDM plots and assessing the list of candidate variants we subtracted a list of wildtype variants compiled from sequencing of the *ekwill* strain plus previously published data (Butler et al., 2015, LaFave et al., 2014, Obholzer et al., 2012). From the prospective candidate mutations in the region of chromosome 23 linked to the mutant phenotype, we filtered for prospective nonsense mutations likely to result in strong loss of function of encoded proteins. The candidate list was further filtered by excluding polymorphisms found in other mutants that we sequenced that derived from the same ENU screen. We designed genotyping assays to test candidate STOP codon-inducing mutation linked to the *ue56* mutant phenotype. We identified a base change (C >T) at position 3034 in the *neurofascin b* coding sequence that was present in all *ue56* sequence reads, and which was predicted to result in a STOP mutation at aa position 1015 of the neurofascin B protein.

#### Mouse Lines

All mouse work conformed to UK legislation (Scientific Procedures Act of 1986), and to University of Edinburgh Ethical Review Committee policy. The generation of *Nfasc*^*-/-*^ mice and Caspr^-/-^ mice has been previously described (Gollan et al., 2003, Sherman et al., 2005). Transgenic mice expressing Nfasc186 with a C-terminal FLAG tag sequence under the control of the Thy1.2 promoter has been described (Zonta et al., 2011) and are referred to here as *Nfasc186*. These mice were interbred with *Nfasc*^*+/-*^ to generate *Nfasc*^*-/-*^*/Nfasc186* mice. All mice were backcrossed to a C57BL/6 background for at least 10 generations.

### Method Details

#### Cloning of *Nfascb* cDNA

To clone wildtype *nfascb* cDNA we carried out PCR with high-fidelity DNA polymerase Phusion (Neb) from a pool of wildtype zebrafish total cDNA (reverse-transcribed from total mRNA extracted from AB 5dpf zebrafish). We used forward primer 5′-ACAAAATTACACCAAAACGCTGT-3′ (which binds 6 bp upstream of the predicted start codon) and reverse primer 5′-AGCCAGTCTGACAGATTAAATGGA-3′ (which binds 45bp downstream of the predicted stop codon), designed based on NCBI transcript XM_005162102 (note that the forward primer anneals to a region currently predicted to be intronic, but previously annotated as an exon). This PCR amplified a 3750 bp cDNA product, which we purified and TOPO-cloned (using the zero Blunt™ TOPO™ PCR Cloning Kit, Thermo Fisher Scientific,450245) to generate pCRII-nfascb. We sequenced four pCRII-nfascb clones and in all we identified a complete *nfascb* ORF of 3501 bp that matched that of *nfascb* predicted transcript variant ×1 mRNA annotated in NCBI (XM_005162102). The sequence was submitted and published in GenBank at ncbi.nlm.nih.gov with the accession number GenBank: MK070493.

#### Rescue of the *nfascb*^*ue56*^ Mutant Phenotype

For in vitro transcription of *nfascb* full length mRNA, *nfascb* cDNA was cloned from pCRII-nfascb into the pCS2+ vector via restriction digest with EcoRI followed by T4 (NEB Biolabs, M0202S) ligation. Full length wildtype *nfascb* mRNA was synthesised from *nfascb* cDNA using the Sp6 mMessage mMachine Kit (Invitrogen, 10391175) and the RNA cleaned up using the RNAeasy Mini Kit (Qiagen, 74104). RNA concentration was measured with Nanodrop One/One (Thermo Fisher Scientific) visualised on an agarose gel.

To test whether full-length wildtype *nfascb* mRNA would rescue the *nfascb*^*ue56*^ mutant phenotype, F1 progeny from Tg(mbp:EGFP-CAAX), *nfascb*^*ue56*/+^ parents were injected with 700-pg synthetic *nfascb* mRNA at the one-cell-stage. Embryos were imaged at 5dpf and then genotyped for homozygosity of the *ue56* mutation.

#### Morpholino Experiments

To deplete Neurofascin B in embryos, 3ng/μl of ATG- blocking morpholino with the sequence TGACAGGAATCCTCCAACACTTCAT was injected at the single cell stage into Tg(mbp:EGFP-CAAX) for whole spinal cord analysis or Tg(sox10:KaltA4), Tg(UAS:mem-GFP) embryos for time lapse analyses. As controls, siblings from the same clutch were injected with the widely used negative control morpholino sequence CCTCTTACCTCAGTTACAATTTATA, that targets a human beta-globin intron (https://www.gene-tools.com/content/negative-control-morpholino-oligos). Animals that showed any abnormal morphology were excluded from the analyses.

#### Genotyping

To genotype *nfascb*^*ue56*^ mutant, heterozygous, and wildtype animals, DNA surrounding the location of the mutation was amplified in a standard PCR reaction using the following primers: 5′-TTGCATGCCTGAGCAGAATA-3′ and 5′-TACTTTCCTCCTCGGCTCCT-3′.

The 143 bp PCR product was then digested with DpnII. Wildtype products were digested into 27 bp and 116 bp fragments, while mutant sequence remained uncut. The PCR products were resolved on a 3% gel.

Genotyping of *caspr*^*sa12772*^ mutant, heterozygous and wildtype animals was carried out by amplifying DNA spanning the mutation using the following primers: 5′-TTTCACACCAACAACATGGAA-3′ and 5′-GGGAAGGGTGGATGGAATAA-3. The 803-bp product was then digested with DdeI. Mutant products were digested into 371 bp and 432 bp fragments, while wildtype sequence remained uncut.

#### Generation of UAS:nfascb-EGFP-pA

To fuse EGFP to the *nfascb* C-terminus, we used recombinant PCR. Using Phusion DNA polymerase, we amplified *nfascb* cDNA using forward primer 5′-GGGGACAAGTTTGTACAAAAAAGCAGGCTGCCACCATGAAGTGTTGGAGGATTCCTGTC-3′, which adds an attB1 sequence and Kozak just before the *nfascb* ORF (which is underlined), and reverse primer 5′-CTCGCCCTTGCTCACCATAGCAAAAGAGTAGATGGCCACAGGAGATGTGGGCTCAGAG-3′, which adds the first 18 bp of the EGFP coding sequence immediately downstream of the penultimate codon of the *nfascb* coding sequence (underlined), excluding its stop codon. In parallel we amplified the EGFP cDNA using forward primer 5′-GCCATCTACTCTTTTGCTATGGTGAGCAAGGGCGAGGAGCTGTTCACCGGGGTGG-3′, which adds the last 18 bp of *nfascb* (prior to the stop codon) upstream of the EGFP coding sequence (underlined); and reverse primer 5′-GGGGACCACTTTGTACAAGAAAGCTGGGTTTACTTGTACAGCTCGTCCATGCC-3′, which adds an attB2R site just downstream of stop codon in the EGFP coding sequence (underlined). We purified these primary PCR products and used equimolar amounts of both as template for the secondary recombinant PCR, using only the attB-containing primers. We then used BP Clonase II to recombine this secondary PCR product with pDONRP4-P1R (plasmid #219 from the tol2kit ((Kwan et al., 2007), http://tol2kit.genetics.utah.edu/index.php/Main_Page) to generate a Gateway-compatible middle-entry vector pME-nfascbEGFP, whose sequence we verified by Sanger sequencing. We then recombined a 5′-entry vector containing 10 UAS repeats (plasmid #327 from the tol2kit (Kwan et al., 2007), ME-nfascb-EGFP, a 3′-entry vector containing a poly adenylation sequence (plasmid #302 from the tol2kit) and destination vector pDestTol2pA2 (plasmid #394 from the tol2kit) using LR Clonase II Plus to generate UAS:nfascb-EGFP-pA.

#### Generation of nfascB-EGFP-UAS-mCherry

We used primers 5′- GGGGACAACTTTGTATAGAAAAGTTGAAAAAACCTCCCACACCTCCCCC-3′ (containing an attB4 site downstream of the polyadenylation sequence) and 5′- GGGGACTGCTTTTTTGTACAAACTTGGCCACCATGAAGTGTTGGAGGATTCCTGTC-3′ (which adds an attB1R site upstream of the *nfascb* coding sequence) to generate 5′-entry vector p5E-nfascB-EGFP-pA, and recombined it using LR Clonase II Plus with middle-entry vector pME-UAS (containing 5 UAS repeats flanked by an E1b minimal promoter in opposite orientations in a Janus configuration (Kwan et al., 2007)), 3′-entry vector p3E-mCherry (plasmid #386 from the tol2kit) and pDestTol2pA2 to generate the plasmid neurofascin B-EGFP-UAS-mCherry, which allows expression of both Nfasc B-EGFP and cytoplasmic mCherry in the same cells, under control of Gal4-regulated UAS sequences.

#### RT-PCR

To test whether *nfascb* mRNA was present in *nfascb*^*ue56*^ homozygous mutants, total RNA was isolated from pooled embryos sorted at 5dpf for the *nfascb*^*ue56*^ mutant phenotype or wildtype embryos, and reverse transcribed using the AccuScript High-Fidelity 1st Strand cDNA Synthesis Kit (Agilent, 200820). The presence of *nfascb* transcript within the cDNA pool of *nfascb*^*ue56*^ versus wildtype embryos was then tested by PCR amplification of a 796 bp fragment using the primer Nfascb_179-795_RT_F 5′-TCCAGTCTTCACATGGACGC-3′ and Nfascb_179-795_RT_R 5′-CGGAACCGATTTGACCCTGA-3′ that bind 179 bp after the start codon of *nfascb*. Reverse transcription of part of *mbpX3* (NCBI RefSeq XM_002665557.4) mRNA was used as a positive control using the primers mbpX3_RT_F 5′-AGAAAGGGAAAGAGACCCCAC-3′ and mbpX3_RT_R 5′-GATCGGCTTTCTCCCAGGTT-3′, and no added reverse transcriptase before PCR amplification as negative control for genomic DNA contamination.

#### FACS and RT-PCR

To perform RT-PCR on total mRNA isolated from only neurons or myelinating glia cells (oligodendrocytes and Schwann cells), cells were fluorescently sorted from embryos expressing Tg(NBT:DsRed) which labels neurons or Tg(mbp:nlsEGFP) which labels myelinating glia cells. 400 embryos for each group were homogenised and incubated for 5 minutes in Ca^2+^ -free Ringer’s solution (116mM NaCl, 2.6mM KCL, 5mM HEPES,pH7) and de-yolked by pipetting (P200). Cell suspensions were then incubated with trypsin-EDTA (0.25% trypsin, 1mM EDTA, pH8, PBS) and Liberase (1:20, 100mg/ml; Roche, RE05401119001) for 15 minutes at 28.5°, while homogenised every 5 minutes with a P1000 pipette. 200–400μl of suspension solution (DMEM, may add: 1% calf serum/NGS, 0.8mM CaCl2,50U/ml penicillin, 0.05mg/ml streptomycin, PBS) + 30% NGS was added and the cell suspension then passed through a 40-μm cell strainer (Falcon, 352340 or 352235). The cells were then centrifuged for 5 min at 350g at 4°C, the supernatant was removed leaving 100μl of cell suspension, and 1ml of chilled suspension solution was added to the cells and the suspension briefly vortexed. This step can be repeated 2–3 times, passing the freshly suspended cells though a 40-μm cell strainer before centrifugation. Finally, the cells were suspended in PBS and transferred to a FACS tube on ice.

Cells were then sorted on a fluorescent cell sorter (Aria Fusion) for DSRed or GFP expression and sorted in into 1ml of ice cold media A (15 mM Hepes (Gibco 15630-056), 25 mM D-glucose (Sigma G8644-100ML) in HBSS 1X (Gibco 14170-088)). Between 10000-20000 myelinating glial cells and 70000–100000 neuronal cells were typically isolated.

Immediately after sorting, RNA was extracted from cell fractions using the RNeasy Plus Micro kit (Qiagen, 74034) and reverse transcribed using the Superscript II reverse transcriptase (Invitrogen, 10635003) to obtain yields of 1–1.2μg of cDNA. PCR was then performed on neuron and myelinating glial cDNA pools using primers nfasc_FACS-RT_F 5′-GGCCCTCCTAAACCAGACAC-3′, nfasc_FACS-RT_R 5′-ATGGGTTTGAAGCGTTGCAC-3′ and caspr_FACS-RT_F 5′-TTTCCGGAAGAACCGTCTGG-3′, caspr_FACS-RT_ R 5′-CTTCCCCCTTGTAGCCTGTG-3′ to test for expression in both cell types, and rbfox3a_FACS-RT_F 5′-AGGGACCAGCAGCTTAACAC-3′, rbfox3a_FACS-RT_R 5′-GCGACTGTAACCTCCTCTGT-3′ and myrf_FACS-RT_F 5′-AATCGTTCTGGGGAACTCGG-3′, myrf_FACS-RT_R 5′-GATCGTTAGCTTGCTGGGGT-3′ as controls for purity of neuronal and glia cell fractions, respectively. *rbfox3a* is the zebrafish ortholog to pan-neuronal mammalian NeuN gene (ZFIN:ZDB-GENE-111213-1).

#### Transmission Electron Microscopy

Tissue was prepared for TEM as previously described (Karttunen et al., 2017). Briefly, zebrafish embryos were terminally anaesthetised in tricaine and incubated, with microwave stimulation, first in primary fixative (4% paraformaldehyde + 2% glutaraldehyde in 0.1M sodium cacodylate buffer) and then in secondary fixative (2% osmium tetroxide in 0.1M sodium cacodylate/imidazole buffer). Samples were then stained en bloc with a saturated uranyl acetate solution and dehydrated in an ethanol series and acetone, both with microwave stimulation. Samples were embedded in EMbed-812 resin (Electron Microscopy Sciences) and sectioned using a Reichert Jung Ultracut Microtome. Sections were cut at comparable somite levels by inspection of blocks under a dissection microscope and stained in uranyl acetate and Sato lead stain. TEM images were taken with a Phillips CM120 Biotwin TEM. The Photomerge tool in Adobe Photoshop was used to automate image registration and tiling. To assess axon diameter and g-ratio, axonal areas were measured in ImageJ with and without their myelin sheath, and diameters (d_axon_ and d_axon+myelin_, respectively) calculated from the obtained values. G-ratios were calculated by dividing axon diameter, d_axon_, by the axon + myelin diameter, d_axon+myelin_.

#### Single Cell Labeling

To mosaically label oligodendrocytes we injected fertilized eggs with 1.5nl of 15ng/μl pTol2-mbp:mCherry-CAAX plasmid DNA and 50ng/μl *tol2* transposase mRNA. Animals were screened at 5dpf for isolated labelled oligodendrocytes and genotyped after imaging.

#### Transgenic Cell-Type Specific Rescue

To express Nfascb-GFP in individual oligodendrocytes in a *nfascb*^*ue56*^ mutant background, the *nfascb*^*ue56*^ allele was crossed into the Tg(sox10:KalTA4) and Tg(ClaudinK:Gal4) lines. We injected F1 progeny from Tg(sox10:KalTA4) or Tg(ClaudinK:Gal4), *nfascb*^*ue56/+*^ crosses with 1.5nL of 15ng/μL pTol2- UAS:nfascb-EGFP-pA or nfascb-EGFP-UAS-mCherry plasmid DNA and 50ng/μl *tol2* transposase mRNA. Embryos were screened for isolated labelled oligodendrocytes at 5dpf and genotyped for homozygosity of the *ue56* mutation after imaging.

#### Live Imaging

For live imaging by confocal microscopy, embryos were anaesthetised with 600μM tricaine and mounted in 1.5% low melting point agarose. Z stacks were acquired using a Zeiss LSM880 confocal microscope equipped with an Airyscan Fast module, using a 20× objective (Zeiss Plan-Apochromat 20× dry, NA = 0.8). Z stacks were acquired with an optimal z-step according to the experiment, e.g. to image through the entirety of a single cell, or the whole spinal cord.

For time-lapse imaging, Tg(sox10:KalTA4,UAS:mGFP) at 2.5 dpf or Tg(mbp:EGFP-CAAX), embryos at 3.5dpf were anaesthetised with 600μM tricaine and mounted in 1.5% low melting point agarose in plastic petri dishes and immersed in E3 medium. Z stacks were again acquired using a Zeiss LSM880 confocal microscope equipped with the Airyscan Fast module and a piezo z-drive, which allowed rapid acquisition of confocal z-stacks. Z stacks were acquired with a z-step between 0.5-1.5 μm and spanned the whole extent of the spinal cord (in the z-axis) in order to cover any drift in z-direction of the embryo during imaging. Z stacks were acquired at 2.5-minute intervals for initial investigation of wildtype myelination and at 10 min to 12 min intervals for experimental comparison of control and *nfascb*-deficient animals. Tg(sox10:KalTA4,UAS:mGFP) control-injected embryos were mounted in the same petri dishes as *nfascb* morpholino injected animals to allow concomitant imaging; similarly Tg(mbp:EGFP-CAAX), *nfascb*^*ue56*^ or Tg(mbp:EGFP-CAAX), *caspr*^*sa12772*^ mutant embryos were imaged alongside their siblings to control for any session-specific variability that might have arisen. The temperature throughout imaging was monitored at 26–28.5°C. Larvae were checked for good blood circulation and general health prior to imaging and genotyped where necessary after imaging.

Typically, only one cell per animal was imaged; when more than one cell per animal was imaged, data were averaged per individual animal. All images and movies represent a lateral view of the spinal cord, anterior to the left and dorsal on top.

#### Image Processing and Analysis

Figure panels were prepared using Fiji and Adobe Photoshop CS6. For figures, maximum-intensity projections of Z stacks were made using Fiji, and a representative x-y area was cropped. All zebrafish images and movies represent a lateral view of the spinal cord, anterior to the left and dorsal on top. For most images, processing using Fiji or Adobe Photoshop included only global change of brightness and contrast; further processing and analysis is as follows.

Raw image and time lapse files were processed using the Zen (black) software after acquisition and then further analysed in Fiji. Time lapse movies were additionally processed using the Zen blue software for time alignment to correct for movement of the embryo during imaging. To single out individual cells or areas in time lapse movies, the original stack was duplicated from a ROI over time and if necessary, cropped in z to eliminate background, or overlaying other cells.

Analysis of cellular attributes at one time point or dynamically over time, such as number of myelin sheaths per cell, number of myelinated cell bodies per cell, myelin sheath length per cell; as well as sheath formation, growth and retraction over time, and cell body myelination, was measured manually using Fiji tools in 3D and maximum intensity projections on z-stacks of one time point and on maximum intensity projections over time for time lapse data, and then quantified in Excel. Fiji counting tools were used to ensure that each sheath or myelinated cell body per oligodendrocyte was only counted once and if not obvious due to strong phenotypic nature, all counts and measurements were performed blinded. Time of sheath formation and sheath growth was calculated from sheath length measurements in every frame. Only myelin-shaped cellular profiles that were stable on axons for greater than 30 minutes were considered as sheaths.

#### Mouse Tissue Preparation and Immunohistochemistry

Spinal cord tissue was collected from P30 wildtype and *Nfasc*^*-/-*^*/Nfasc186* mice after transcardiac perfusion with 4% paraformaldehyde in 0.1-M phosphate buffer pH 7.4 and post-fixed for 1hrs. For *Caspr*^*-/-*^ mice, spinal cord was collected at P23 for both wildtype and mutant mice. The sex of mice was not assessed. After fixation, spinal cord tissue was washed with phosphate buffered saline (PBS) and then cryoprotected in 25% sucrose in PBS at 4°C. Tissue samples were oriented in O.C.T. embedding matrix compound (CellPath Ltd) and frozen with liquid nitrogen cooled isopentane. The blocks were then stored at -80°C until use. Coronal sections (25 μm) were cut from the cervical spinal cord (C5-C8) using a Leica CM3050 cryostat and collected on SuperFrost Slides (Thermo Scientific). Sections were dried and stored at -20°C until use.

Teased fibers were obtained from the ventral funiculus of the cervical spinal cord using acupuncture needles as described (Jarjour and Sherman, 2019).

Cryostat sections or teased fibers were blocked in blocking solution (5% fish skin gelatine (Sigma), 0.2% Triton X-100 in PBS) for 1 hrs at room temperature. Primary antibodies were diluted in the same buffer and incubated overnight at room temperature in humidified chambers, followed by PBS washes. AlexaFluor (1:1000) or FITC (1:200)-conjugated secondary antibodies were applied for 1.5 hrs (Molecular Probes, Jackson ImmunoResearch, Southern Biotech). Primary antibodies were used at the following dilutions: rabbit anti-MBP (1:1000, (Tait et al., 2000) , rabbit anti-Nfasc186 (MNF2 1:1000, (Vouyiouklis and Brophy, 1993)), mouse anti-Caspr (1:50, kind gift of Dr Matthew Rasband, Baylor College of Medicine,TX), mouse anti-Kv1.1 (1:200, UC Davis/NIH NeuroMab Facility, K36/15), mouse anti-NeuN (1:500, Millipore, MAB377), mouse anti-NF-200 (1:2000, Sigma N0142). Samples were mounted in Vectashield (Vector Laboratories).

#### Mouse Spinal Cord Image Acquisition and Analysis

Images of the dorsal horn were acquired using a Zeiss AxioImager Z1 microscope equipped with an Apotome2 structured illumination unit and a 20× objective (Zeiss Plan-Apochromat 20× dry, NA = 0.8). Images of neuronal cell bodies were acquired using a confocal microscope Zeiss LSM880 equipped with Airyscanner and a 20× objective (Zeiss Plan-Apochromat 20× dry, NA = 0.8). Z stacks were acquired with a z-step between 0.8 μm according to the experiment. The incidence of wrapped cell bodies (as determined by tight apposition of NeuN+ cell bodies with MBP throughout individual planes in the z stack) was quantified blinded. The region of the dorsal horn containing NeuN+ cell bodies (inside the dotted lines in the images) from 10 cervical spinal cord (C5 – C8) hemi-sections was analysed per mouse (n=5). Internodal length measurements were obtained as the distance between two nodes identified by Nfasc186 immunolabelling. A minimum of 50 internodes were measured per mouse (n=5) using Fiji (Schindelin et al., 2012).

### Quantification and Statistical Analyses

All graphs and statistical tests were carried out using GraphPad Prism. Data was tested for normal distribution using D’Agostino-Pearson omnibus test and tested for significance between more than two groups with one-way ANOVA or Kruskal-Wallis test if data was normally distributed or not, respectively. We also compared between groups using a two-tailed unpaired Student’s t test or using the Mann-Whitney U test. We considered a difference significant when p < 0.05. Throughout the figures, we indicate p values as follows: non-significant i.e. p > 0.05 ‘ns,’, p < 0.05, ‘^∗^’ p < 0.01, ‘^∗∗^’ p < 0.001‘^∗∗∗^’ p < 0.0001 ‘^∗∗∗∗^’. Indicated p-values are from t-test comparisons in all graphs where datasets passed the normality test; when data was clearly not normally distributed, we indicate the result of the Mann-Whitney U test and data points are displayed with interquartile range rather than standard deviation (SD) or standard error of the mean (SEM). Throughout the figures, error bars indicate either mean ± SD, or SEM where noted. Details of statistical tests used, precise p value and n values for each comparison are detailed in Figure Legends.

### Data and Code Availability

The published article includes all datasets analyzed during this study.

## Supplemental Information

Document S1. Figures S1–S3

Document S2. Article plus Supplemental Information

## References

[bib1] Almeida R.G. (2018). The rules of attraction in central nervous system myelination. Front. Cell. Neurosci..

[bib2] Almeida R.G., Czopka T., ffrench-Constant C., Lyons D.A. (2011). Individual axons regulate the myelinating potential of single oligodendrocytes in vivo. Development.

[bib3] Almeida R.G., Lyons D.A. (2015). Intersectional gene expression in zebrafish using the split KalTA4 system. Zebrafish.

[bib4] Almeida R.G., Lyons D.A. (2017). On myelinated axon plasticity and neuronal circuit formation and function. J. Neurosci..

[bib5] Almeida R.G., Pan S., Cole K.L.H., Williamson J.M., Early J.J., Czopka T., Klingseisen A., Chan J.R., Lyons D.A. (2018). Myelination of neuronal cell bodies when myelin supply exceeds axonal demand. Curr. Biol..

[bib6] Auer F., Vagionitis S., Czopka T. (2018). Evidence for myelin sheath remodeling in the CNS revealed by in vivo imaging. Curr. Biol..

[bib7] Baraban M., Koudelka S., Lyons D.A. (2018). Ca 2+ activity signatures of myelin sheath formation and growth in vivo. Nat. Neurosci..

[bib8] Bazzoni G. (2003). The JAM family of junctional adhesion molecules. Curr. Opin. Cell Biol..

[bib9] Bechler M.E., Byrne L., ffrench-Constant C. (2015). CNS myelin sheath lengths are an intrinsic property of oligodendrocytes. Curr. Biol..

[bib10] Bhat M.A., Rios J.C., Lu Y., Garcia-Fresco G.P., Ching W., St Martin M., Li J., Einheber S., Chesler M., Rosenbluth J. (2001). Axon-glia interactions and the domain organization of myelinated axons requires neurexin IV/Caspr/Paranodin. Neuron.

[bib11] Boyle M.E., Berglund E.O., Murai K.K., Weber L., Peles E., Ranscht B. (2001). Contactin orchestrates assembly of the septate-like junctions at the paranode in myelinated peripheral nerve. Neuron.

[bib12] Brivio V., Faivre-Sarrailh C., Peles E., Sherman D.L., Brophy P.J. (2017). Assembly of CNS nodes of Ranvier in myelinated nerves is promoted by the axon cytoskeleton. Curr. Biol..

[bib13] Butler M.G., Iben J.R., Marsden K.C., Epstein J.A., Granato M., Weinstein B.M. (2015). SNPfisher: tools for probing genetic variation in laboratory-reared zebrafish. Development.

[bib14] Charles P., Tait S., Faivre-Sarrailh C., Barbin G., Gunn-Moore F., Denisenko-Nehrbass N., Guennoc A.M., Girault J.A., Brophy P.J., Lubetzki C. (2002). Neurofascin is a glial receptor for the paranodin/Caspr-contactin axonal complex at the axoglial junction. Curr. Biol..

[bib15] Çolakoğlu G., Bergstrom-Tyrberg U., Berglund E.O., Ranscht B. (2014). Contactin-1 regulates myelination and nodal/paranodal domain organization in the central nervous system. Proc. Natl. Acad. Sci. USA.

[bib16] Collinson J.M., Marshall D., Gillespie C.S., Brophy P.J. (1998). Transient expression of neurofascin by oligodendrocytes at the onset of myelinogenesis: implications for mechanisms of axon-glial interaction. Glia.

[bib17] Czopka T., ffrench-Constant C., Lyons D.A. (2013). Individual oligodendrocytes have only a few hours in which to generate new myelin sheaths in vivo. Dev. Cell.

[bib18] Efthymiou S., Salpietro V., Malintan N., Poncelet M., Kriouile Y., Fortuna S., De Zorzi R., Payne K., Henderson L.B., Cortese A. (2019). Biallelic mutations in neurofascin cause neurodevelopmental impairment and peripheral demyelination. Brain.

[bib19] Einheber S., Zanazzi G., Ching W., Scherer S., Milner T.A., Peles E., Salzer J.L. (1997). The axonal membrane protein Caspr, a homologue of neurexin IV, is a component of the septate-like paranodal junctions that assemble during myelination. J. Cell Biol..

[bib20] Elazar N., Vainshtein A., Golan N., Vijayaragavan B., Schaeren-Wiemers N., Eshed-Eisenbach Y., Peles E. (2019). Axoglial adhesion by Cadm4 regulates CNS myelination. Neuron.

[bib21] Elazar N., Vainshtein A., Rechav K., Tsoory M., Eshed-Eisenbach Y., Peles E. (2019). Coordinated internodal and paranodal adhesion controls accurate myelination by oligodendrocytes. J. Cell Biol..

[bib22] Etxeberria A., Hokanson K.C., Dao D.Q., Mayoral S.R., Mei F., Redmond S.A., Ullian E.M., Chan J.R. (2016). Dynamic modulation of myelination in response to visual stimuli alters optic nerve conduction velocity. J. Neurosci..

[bib23] Ford M.C., Alexandrova O., Cossell L., Stange-Marten A., Sinclair J., Kopp-Scheinpflug C., Pecka M., Attwell D., Grothe B. (2015). Tuning of Ranvier node and internode properties in myelinated axons to adjust action potential timing. Nat. Commun..

[bib24] Goebbels S., Wieser G.L., Pieper A., Spitzer S., Weege B., Yan K., Edgar J.M., Yagensky O., Wichert S.P., Agarwal A. (2017). A neuronal PI(3,4,5)P3-dependent program of oligodendrocyte precursor recruitment and myelination. Nat. Neurosci..

[bib25] Gollan L., Salomon D., Salzer J.L., Peles E. (2003). Caspr regulates the processing of contactin and inhibits its binding to neurofascin. J. Cell Biol..

[bib26] Hill R.A., Li A.M., Grutzendler J. (2018). Lifelong cortical myelin plasticity and age-related degeneration in the live mammalian brain. Nat. Neurosci..

[bib27] Hines J.H., Ravanelli A.M., Schwindt R., Scott E.K., Appel B. (2015). Neuronal activity biases axon selection for myelination in vivo. Nat. Neurosci..

[bib28] Hinman J.D., Peters A., Cabral H., Rosene D.L., Hollander W., Rasband M.N., Abraham C.R. (2006). Age-related molecular reorganization at the node of Ranvier. J. Comp. Neurol..

[bib29] Howell O.W., Palser A., Polito A., Melrose S., Zonta B., Scheiermann C., Vora A.J., Brophy P.J., Reynolds R. (2006). Disruption of neurofascin localization reveals early changes preceding demyelination and remyelination in multiple sclerosis. Brain.

[bib30] Hughes E.G., Orthmann-Murphy J.L., Langseth A.J., Bergles D.E. (2018). Myelin remodeling through experience-dependent oligodendrogenesis in the adult somatosensory cortex. Nat. Neurosci..

[bib31] Jarjour A.A., Sherman D.L. (2019). Teasing of ventral spinal cord White matter fibers for the analysis of central nervous system nodes of Ranvier. Methods Mol. Biol..

[bib32] Karttunen M.J., Czopka T., Goedhart M., Early J.J., Lyons D.A. (2017). Regeneration of myelin sheaths of normal length and thickness in the zebrafish CNS correlates with growth of axons in caliber. PLoS One.

[bib63] Kegel L., Rubio M., Almeida R.G., Benito S., Klingseisen A., Lyons D.A. (2019). Forward genetic screen using zebrafish to identify new genes involved in myelination. Oligodendrocytes, Oligodendrocytes: Methods and Protocols.

[bib33] Kelsh R.N., Brand M., Jiang Y.J., Heisenberg C.P., Lin S., Haffter P., Odenthal J., Mullins M.C., van Eeden F.J., Furutani-Seiki M. (1996). Zebrafish pigmentation mutations and the processes of neural crest development. Development.

[bib34] Kettleborough R.N.W., Busch-Nentwich E.M., Harvey S.A., Dooley C.M., de Bruijn E., van Eeden F., Sealy I., White R.J., Herd C., Nijman I.J. (2013). A systematic genome-wide analysis of zebrafish protein-coding gene function. Nature.

[bib35] Klingseisen A., Lyons D.A. (2017). Axonal regulation of central nervous system myelination: structure and function. Neuroscientist.

[bib36] Koudelka S., Voas M.G., Almeida R.G., Baraban M., Soetaert J., Meyer M.P., Talbot W.S., Lyons D.A. (2016). Individual neuronal subtypes exhibit diversity in CNS myelination mediated by synaptic vesicle release. Curr. Biol..

[bib37] Kwan K.M., Fujimoto E., Grabher C., Mangum B.D., Hardy M.E., Campbell D.S., Parant J.M., Yost H.J., Kanki J.P., Chien C.B. (2007). The Tol2kit: a multisite gateway-based construction kit for Tol2 transposon transgenesis constructs. Dev. Dyn..

[bib38] LaFave M.C., Varshney G.K., Vemulapalli M., Mullikin J.C., Burgess S.M. (2014). A defined zebrafish line for high-throughput genetics and genomics: NHGRI-1. Genetics.

[bib39] Lee S., Leach M.K., Redmond S.A., Chong S.Y.C., Mellon S.H., Tuck S.J., Feng Z.Q., Corey J.M., Chan J.R. (2012). A culture system to study oligodendrocyte myelination processes using engineered nanofibers. Nat. Methods.

[bib40] Low K.J., Stals K., Caswell R., Wakeling M., Clayton-Smith J., Donaldson A., Foulds N., Norman A., Splitt M., Urankar K. (2018). Phenotype of CNTNAP1: a study of patients demonstrating a specific severe congenital hypomyelinating neuropathy with survival beyond infancy. Eur. J. Hum. Genet..

[bib41] Maier O., van der Heide T., Johnson R., de Vries H., Baron W., Hoekstra D. (2006). The function of neurofascin155 in oligodendrocytes is regulated by metalloprotease-mediated cleavage and ectodomain shedding. Exp. Cell Res..

[bib42] Mensch S., Baraban M., Almeida R., Czopka T., Ausborn J., El Manira A., Lyons D.A. (2015). Synaptic vesicle release regulates myelin sheath number of individual oligodendrocytes in vivo. Nat. Neurosci..

[bib43] Minevich G., Park D.S., Blankenberg D., Poole R.J., Hobert O. (2012). CloudMap: A cloud-based pipeline for analysis of mutant genome sequences. Genetics.

[bib44] Mitew S., Gobius I., Fenlon L.R., McDougall S.J., Hawkes D., Xing Y.L., Bujalka H., Gundlach A.L., Richards L.J., Kilpatrick T.J. (2018). Pharmacogenetic stimulation of neuronal activity increases myelination in an axon-specific manner. Nat. Commun..

[bib45] Münzel E.J., Schaefer K., Obirei B., Kremmer E., Burton E.A., Kuscha V., Becker C.G., Brösamle C., Williams A., Becker T. (2012). Claudin k is specifically expressed in cells that form myelin during development of the nervous system and regeneration of the optic nerve in adult zebrafish. Glia.

[bib46] Obholzer N., Swinburne I.A., Schwab E., Nechiporuk A.V., Nicolson T., Megason S.G. (2012). Rapid positional cloning of zebrafish mutations by linkage and homozygosity mapping using whole-genome sequencing. Development.

[bib47] Peri F., Nüsslein-Volhard C. (2008). Live imaging of neuronal degradation by microglia reveals a role for v0-ATPase a1 in phagosomal fusion in vivo. Cell.

[bib48] Pillai A.M., Thaxton C., Pribisko A.L., Cheng J.G., Dupree J.L., Bhat M.A. (2009). Spatiotemporal ablation of myelinating glia-specific neurofascin(Nfasc NF155) in mice reveals gradual loss of paranodal axoglial junctions and concomitant disorganization of axonal domains. J. Neurosci. Res..

[bib49] Redmond S.A., Mei F., Eshed-Eisenbach Y., Osso L.A., Leshkowitz D., Shen Y.-A.A., Kay J.N., Aurrand-Lions M., Lyons D.A., Peles E. (2016). Somatodendritic expression of JAM2 inhibits oligodendrocyte myelination. Neuron.

[bib50] Saab A.S., Nave K.A. (2017). Myelin dynamics: protecting and shaping neuronal functions. Curr. Opin. Neurobiol..

[bib51] Schindelin J., Arganda-Carreras I., Frise E., Kaynig V., Longair M., Pietzsch T., Preibisch S., Rueden C., Saalfeld S., Schmid B. (2012). Fiji: an open-source platform for biological-image analysis. Nat. Methods.

[bib52] Seidl A.H. (2014). Regulation of conduction time along axons. Neuroscience.

[bib53] Sherman D.L., Tait S., Melrose S., Johnson R., Zonta B., Court F.A., Macklin W.B., Meek S., Smith A.J., Cottrell D.F. (2005). Neurofascins are required to establish axonal domains for saltatory conduction. Neuron.

[bib54] Smigiel R., Sherman D.L., Rydzanicz M., Walczak A., Mikolajkow D., Krolak-Olejnik B., Kosińska J., Gasperowicz P., Biernacka A., Stawinski P. (2018). Homozygous mutation in the neurofascin gene affecting the glial isoform of neurofascin causes severe neurodevelopment disorder with hypotonia, amimia and areflexia. Hum. Mol. Genet..

[bib55] Snaidero N., Möbius W., Czopka T., Hekking L.H.P., Mathisen C., Verkleij D., Goebbels S., Edgar J., Merkler D., Lyons D.A. (2014). Myelin membrane wrapping of CNS axons by PI(3,4,5)P3-dependent polarized growth at the inner tongue. Cell.

[bib56] Tait S., Gunn-Moore F., Collinson J.M., Huang J., Lubetzki C., Pedraza L., Sherman D.L., Colman D.R., Brophy P.J. (2000). An oligodendrocyte cell adhesion molecule at the site of assembly of the paranodal axo-glial junction. J. Cell Biol..

[bib57] Voas M.G., Glenn T.D., Raphael A.R., Talbot W.S. (2009). Schwann cells inhibit ectopic clustering of axonal sodium channels. J. Neurosci..

[bib58] Vouyiouklis D.A., Brophy P.J. (1993). Microtubule-associated protein MAP1B expression precedes the morphological differentiation of oligodendrocytes. J. Neurosci. Res..

[bib59] Wake H., Ortiz F.C., Woo D.H., Lee P.R., Angulo M.C., Fields R.D. (2015). Nonsynaptic junctions on myelinating glia promote preferential myelination of electrically active axons. Nat. Commun..

[bib60] Watkins T.A., Emery B., Mulinyawe S., Barres B.A. (2008). Distinct stages of myelination regulated by gamma-secretase and astrocytes in a rapidly myelinating CNS coculture system. Neuron.

[bib61] Zonta B., Desmazieres A., Rinaldi A., Tait S., Sherman D.L., Nolan M.F., Brophy P.J. (2011). A critical role for neurofascin in regulating action potential initiation through maintenance of the axon initial segment. Neuron.

[bib62] Zonta B., Tait S., Melrose S., Anderson H., Harroch S., Higginson J., Sherman D.L., Brophy P.J. (2008). Glial and neuronal isoforms of neurofascin have distinct roles in the assembly of nodes of Ranvier in the central nervous system. J. Cell Biol..

