# Supplementary material for: Oligodendrocyte Neurofascin Independently Regulates Both Myelin Targeting and Sheath Growth in the CNS
Source: Dev Cell. 2019 Dec 16;51(6):730–744.e6. doi: 10.1016/j.devcel.2019.10.016 (PMC6912162; doi:10.1016/j.devcel.2019.10.016)
Supplement: Document S1. Figures S1–S3 [file mmc1.pdf]

Developmental Cell, Volume *51*

Supplemental Information

Oligodendrocyte Neurofascin Independently Regulates Both Myelin Targeting and Sheath Growth in the CNS

Anna Klingseisen, Ana-Maria Ristoiu, Linde Kegel, Diane L. Sherman, Maria Rubio-Brotons, Rafael G. Almeida, Sigrid Koudelka, Silvia K. Benito-Kwiecinski, Richard J. Poole, Peter J. Brophy, and David A. Lyons

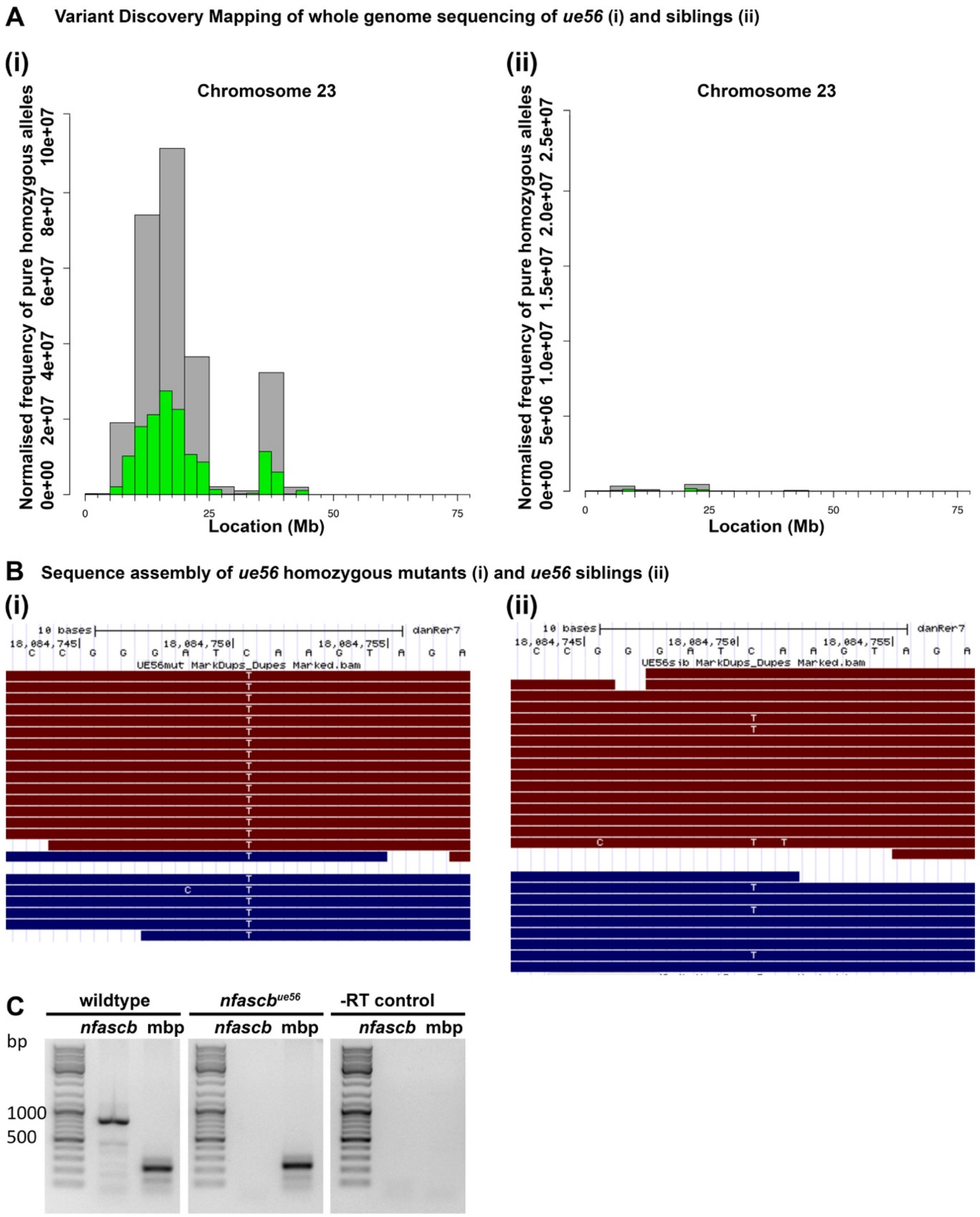

Figure S1. Related to Figure 1. *ue56* disrupts zebrafish *neurofascin b*

A. Variant Discovery Mapping plots from the CloudMap pipeline showing the normalised frequency of pure homozygous variants (allele frequency in recombinant pool = 1.0) in 2.5 Mb (green) or 5 Mb (grey) bins.

B. Sequence reads showing a C to T transition in all *ue56* mutant traces left, and in a subset of sibling traces (combination of wildtype and het animals) left.

C. RT-PCR analyses of *nfascb* and *mbp* expression in wildtype animals. There is essentially no detectable *nfascb* mRNA in *ue56* mutants at 5dpf.

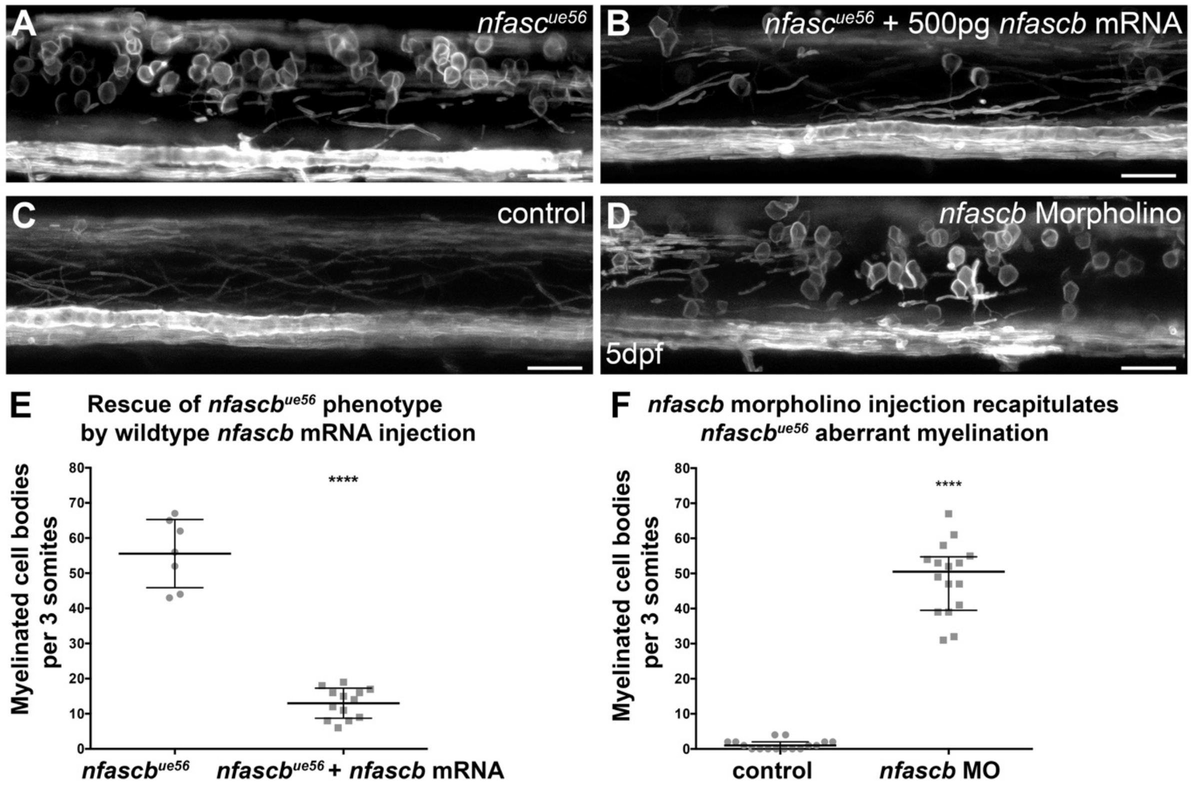

Figure S2. Related to Figure 1. Wildtype *neurofascin b* rescues *ue56* mutants and *neurofascin b* knockdown phenocopies *ue56* mutants

A+B. Confocal images of myelin labelled by Tg(mbp:EGFP-CAAX) in *nfascb*^{ue56} mutants, control injected (A), and injected with wildtype *nfascb* mRNA (B). Scale bar=20µm.

C+D. Confocal images of myelin labelled by Tg(mbp:EGFP-CAAX) in a control injected (C), and antisense Morpholino targeting *nfascb*-injected animal (D). Scale bar=20µm.

E. Number of myelinated cell bodies in *ue56* mutants injected with control solution vs wildtype *nfascb* mRNA (*nfascb*^{ue56} mutants 55.57 ± 9.71 SD, n=7 animals, *nfascb*^{ue56} mutants + n*fascb* mRNA mean 13.00 ± 4.28 SD, n=13 animals, p < 0.0001, t-test).

F. Number of myelinated cell bodies in wildtype animals injected with control solution vs antisense Morpholino targeting *nfascb* (wildtype median =1, 25th percentile 0.0, 75th percentile 2.0, n=16 animals, *nfascb* Morpholino median 50.50, 25th percentile 39.50, 75th percentile 54.75, n=16 animals, p < 0.0001, Mann Whitney test).

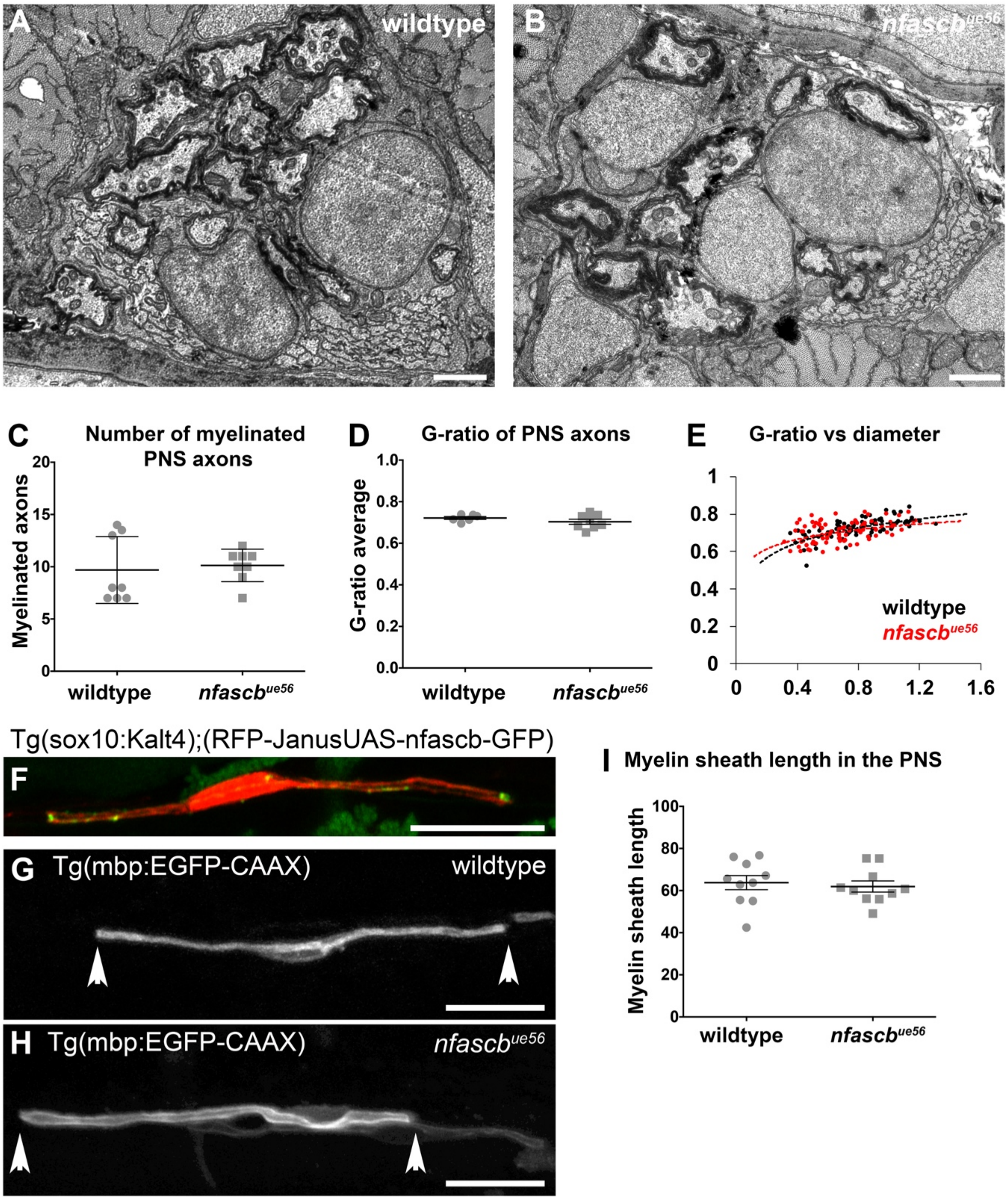

Figure S3. Related to Figures 2 and 4. Myelination of the posterior lateral line nerve is normal at 5 dpf in *nfascb*^{ue56} mutants.

A+B. TEM images of transverse sections cut through the posterior lateral line nerve of wildtype (**A**) and *nfascb*^{ue56} mutant (**B**) animals. Scale bars = 1 µm.

C. Number of myelinated axons in wildtype and *nfascb*^{ue56} mutants (wildtype mean 9.69 ± 3.20 SD, n= 6 animals and *nfascb*^{ue56} mutant mean 10.13 ± 1.16 SD, n=7 animals, p= 0.7328, t-test).

D. Average g-ratio per animal in wildtype and *nfascb*^{ue56} mutants (wildtype mean 0.72 ± 0.01 SEM, n= 6 animals and *nfascb*^{ue56} mutant mean 0.70 ± 0.01 SEM, n=7 animals, p= 0.2542, t-test).

E. Assessment of g-ratio plotted with respect to axon caliber for all myelianted axons measured in wildtype and *nfascb*^{ue56} mutants.
F. Confocal image of a single Schwann cell expressing a cytoplasmic red fluorescent protein and Nfasc B-GFP. Scale bar = 20µm.
G+H. Confocal images of single Schwann cells in Tg(mbp:EGFP-CAAX) wildtype (**G**) and *nfascb*^{ue56} mutant (**H**) animals. Arrowheads point to the ends of the myelin sheaths. Scale bars = 20 µm.
I. Average length of myelin sheaths made by Schwann cells in wildtype and *nfascb*^{ue56} mutants (wildtype mean 63.78 ± 3.35 SEM, n= 10 animals and *nfascb*^{ue56} mutant mean 61.92 ± 2.64 SEM, n=10 animals, p= 0.6690, t-test).

Video S1 (relates to Figure 5).
Time-lapse movie of oligodendrocyte in Tg(sox10KalTA4, UAS mEGFP) wildtype animal. Individual myelinating processes can engage in various aspects of myelination concomitantly, from forming new sheaths, growing myelin sheaths at different rates, and retracting from axons or cell bodies.

Video S2 (relates to Figure 5).
Time-lapse movie of oligodendrocyte in Tg(sox10KalTA4, UAS mEGFP) control-injected animal. Oligodendrocytes form their myelin sheaths in a short dynamic period of a few hours.

Video S3 (relates to Figure 5).
Time-lapse movie of oligodendrocyte in Tg(sox10KalTA4, UAS mEGFP) *nfascb* MO-injected animal. Oligodendrocytes form their myelin sheaths in a short dynamic period of a few hours and also myelinated cell bodies during the same time.

Video S4 (relates to Figure 5).
Time-lapse movie of oligodendrocyte in Tg(sox10KalTA4, UAS mEGFP) *nfascb* MO-injected animal. A myelinating process contacts a cell body and extends a sheet-like protrusion around the axon to enwrap it.

Video S5 (relates to Figure 5).
Time-lapse movie of oligodendrocyte in Tg(sox10KalTA4, UAS mEGFP) control-injected animal. A myelinating process contacts a cell body, begins to ensheath it, but subsequently retracts.

Video S6 (relates to Figure 6).
Time-lapse movie of a myelin sheath elongating quickly in a Tg(sox10KalTA4, UAS mEGFP) control-injected animal.

Video S7 (relates to Figure 6).
Time-lapse movie of a myelin sheath elongating slowly in a Tg(sox10KalTA4, UAS mEGFP) *nfascb* MO -injected animal.

Video S8 (relates to Figure 6).
Time-lapse movie of a myelin sheath shrinking in a Tg(sox10KalTA4, UAS mEGFP) *nfascb* MO-injected animal.

Video S9 (relates to Figure 6)
Time-lapse movie of a myelin sheath growing in a Tg(mbp:EGFP-CAAX) wildtype animal.

Video S10 (relates to Figure 6)
Time-lapse movie of a myelin sheath growing in a Tg(mbp:EGFP-CAAX) $nfascb^{ue56}$ animal.

Video S11 (relates to Figure 6)
Time-lapse movie of myelination of a cell body in a Tg(mbp:EGFP-CAAX) $nfascb^{ue56}$ animal.
